# Development of Fe_3_O_4_ core–TiO_2_ shell nanocomposites and nanoconjugates as a foundation for neuroblastoma radiosensitization

**DOI:** 10.1186/s12645-021-00081-z

**Published:** 2021-05-14

**Authors:** William Liu, Salida Mirzoeva, Ye Yuan, Junjing Deng, Si Chen, Barry Lai, Stefan Vogt, Karna Shah, Rahul Shroff, Reiner Bleher, Qiaoling Jin, Nghia Vo, Remon Bazak, Carissa Ritner, Stanley Gutionov, Sumita Raha, Julia Sedlmair, Carol Hirschmugl, Chris Jacobsen, Tatjana Paunesku, John Kalapurkal, Gayle E. Woloschak

**Affiliations:** 1 Department of Radiation Oncology, Northwestern University, Chicago, IL 60611 USA; 2 Department of Physics and Astronomy, Northwestern University, Evanston, IL 60208 USA; 3 X-Ray Science Division, Argonne National Laboratory, Argonne, IL 60439 USA; 4 Chemistry of Life Processes Institute, Northwestern University, Evanston, IL 60208 USA; 5 Diamond Light Source Ltd, Harwell Science and Innovation Campus, Didcot, OX11 0DE UK; 6 Synchrotron Radiation Center, 3731 Schneider Drive, Stoughton, WI 53589-3097 USA; 7 Physics Department, University of Wisconsin-Milwaukee, Milwaukee, WI 53211 USA; 8 Department of Otorhinolaryngology, Faculty of Medicine, University of Alexandria, Alexandria, Egypt

**Keywords:** Nanocomposites, Nanoconjugates, Iron oxide core nanoparticles, Titanium dioxide shell nanoparticles, Neuroblastoma, Radiosensitization

## Abstract

**Background:**

Neuroblastoma is the most common extracranial solid malignancy in childhood which, despite the current progress in radiotherapy and chemotherapy protocols, still has a high mortality rate in high risk tumors. Nanomedicine offers exciting and unexploited opportunities to overcome the shortcomings of conventional medicine. The photocatalytic properties of Fe_3_O_4_ core-TiO_2_ shell nanocomposites and their potential for cell specific targeting suggest that nanoconstructs produced using Fe_3_O_4_ core-TiO_2_ shell nanocomposites could be used to enhance radiation effects in neuroblastoma. In this study, we evaluated bare, metaiodobenzylguanidine (MIBG) and 3,4-Dihydroxyphenylacetic acid (DOPAC) coated Fe_3_O_4_@TiO_2_ as potential radiosensitizers for neuroblastoma in vitro.

**Results:**

The uptake of bare and MIBG coated nanocomposites modestly sensitized neuroblastoma cells to ionizing radiation. Conversely, cells exposed to DOPAC coated nanocomposites exhibited a five-fold enhanced sensitivity to radiation, increased numbers of radiation induced DNA double-strand breaks, and apoptotic cell death. The addition of a peptide mimic of the epidermal growth factor (EGF) to nanoconjugates coated with MIBG altered their intracellular distribution. Cryo X-ray fluorescence microscopy tomography of frozen hydrated cells treated with these nanoconjugates revealed cytoplasmic as well as nuclear distribution of the nanoconstructs.

**Conclusions:**

The intracellular distribution pattern of different nanoconjugates used in this study was different for different nanoconjugate surface molecules. Cells exposed to DOPAC covered nanoconjugates showed the smallest nanoconjugate uptake, with the most prominent pattern of large intracellular aggregates. Interestingly, cells treated with this nanoconjugate also showed the most pronounced radiosensitization effect in combination with the external beam x-ray irradiation. Further studies are necessary to evaluate mechanistic basis for this increased radiosensitization effect. Preliminary studies with the nanoparticles carrying an EGF mimicking peptide showed that this approach to targeting could perhaps be combined with a different approach to radiosensitization – use of nanoconjugates in combination with the radioactive iodine. Much additional work will be necessary in order to evaluate possible benefits of targeted nanoconjugates carrying radionuclides.

**Graphic abstract:**

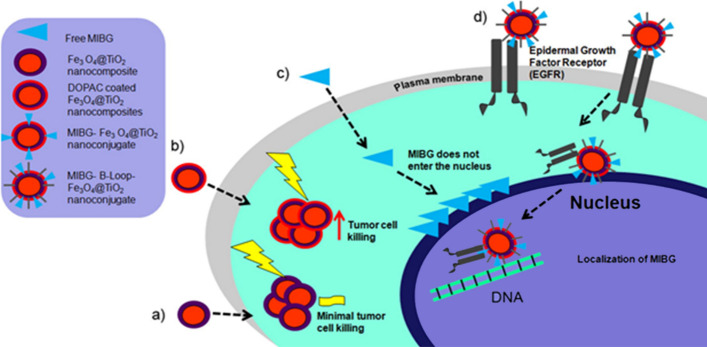

**Supplementary Information:**

The online version contains supplementary material available at 10.1186/s12645-021-00081-z.

## Background

Neuroblastoma is the most common extracranial solid malignancy among children under 15 years of age (Baade et al. 2010; Gatta et al. 2012; Kacar et al. 2013; Stiller 2004; Ward et al. 2014). It arises within the sympathetic nervous system from primordial neural crest cells of sympaticoadrenal lineage. Neuroblastoma is a heterogeneous tumor with a highly variable clinical behavior. Although low- to intermediate-risk tumors tend to have a favorable prognosis, high-risk tumors often exhibit less than 50% overall survival rate (Maris et al. 2007). The variable clinical outcome, which occasionally mounts to inexorable progression despite intensive therapy, reflects the genetic heterogeneity of these tumors (Aygun 2018). The most consistent genetic abnormality associated with adverse outcomes is genomic amplification of N-myc proto-oncogene (MYCN), which occurs in nearly 20% of primary tumors (Maris et al. 2007). Neuroblastoma is notorious for relapse, which occurs in up to 60% of cases and is often refractive to most currently available treatment modalities (Maris 2010). Primary neuroblastomas often have a wild-type p53 which in combination with MYCN, often results in treatment induced apoptosis (Yoshida 2018). However, upon relapse, neuroblastomas often become p53-negative which renders these tumors resistant to therapy (Huang and Weiss 2013). Gene sequencing of neuroblastoma samples has revealed that the genetic makeup of up to 78% of relapsed neuroblastomas harbors new genetic mutations in the RAS–mitogen-activated protein kinase pathway (RAS–MAPK) and novel MYCN amplifications (Eleveld et al. 2015; Schramm et al. 2015). The current standard of care for neuroblastoma entails multiple treatment modalities, all of which have a deleterious effect on health (Maris 2010). Even targeted therapies such as radioactive metaiodobenzylguanidine (MIBG) carry with it numerous treatment-related hurdles ranging from myelosuppression as the dose-limiting toxicity, to difficulties in isolating young radio-iodine treated children which is essential for protection of caregivers (DuBois and Matthay 2008; Matthay et al. 2012,2007). New approaches with targeted therapies are under investigation; however, few are as efficient for relapsed neuroblastoma as treatment with radioactive MIBG. It should be noted that there are two types of radioactive iodine that can be used for preparation of MIBG. Typically, ^131^I-MIBG is used for therapy, while ^123^I-MIBG is used for diagnosis by scintigraphy (Nakatani et al. 2002). ^131^I is a β-emitter, with an effective tissue penetration of 2 cm and it is most efficient when used for treatment of larger tumors where uptake by some of the cells kills the neighboring tumor cells (Cunningham et al. 1998). For micrometastases below 1 mm, the dose of ^131^I-MIBG-derived radiation absorbed by the tumor vs. surrounding tissue declines significantly (Sisson et al. 1990). On the other hand, ^125^I is a low-energy gamma emitter and an Auger electron emitter, with a maximum effective range of roughly 30 µm (Weber et al. 1996). Consequently, ^125^I-MIBG is more effective in killing smaller metastases as shown in an animal model (Rutgers et al. 2000). Our work presented here is motivated by the possibility to use the short-range emitting ^125^I-MIBG in combination with nanoparticles that may alter subcellular distribution of this agent and make it more potent. At the same time, we wished to explore whether the nanoparticles themselves had the capacity to increase sensitivity to radiation.

Nanotechnology provides a myriad of encouraging targeting opportunities to tackle current limitations in conventional treatment modalities (Bazak et al. 2014,2015). Fe_3_O_4_ core–TiO_2_ shell (Fe_3_O_4_@TiO_2_) nanocomposites are unique in having surface reactivity and photocatalytic properties that render them an attractive platform for inducing controlled targeted cytotoxicity and DNA scission in neoplastic cells (Bazak et al. 2013; Yuan et al. 2013). The photocatalytic properties of Fe_3_O_4_@TiO_2_ nanocomposites are based on the ability of TiO_2_ shell to produce reactive oxygen species (ROS) when exposed to photons with energies greater than 3.2 eV (Blake et al. 1999). These ROS interact with adjacent intracellular structures to induce direct deoxyribonucleic acid (DNA) damage (Cooke et al. 2003), mitochondrial ROS-induced ROS release (Zorov et al. 2006), and ultimately cell death (Ryter et al. 2007). Owing to the unique physico-chemical characteristics of Fe_3_O_4_@TiO_2_ nanocomposites, they have been explored in several contexts. Arora and others (Arora et al. 2012) utilized Fe_3_O_4_@TiO_2_ nanoconstructs for intracellular delivery of doxorubicin circumventing drug resistance in ovarian cancer cell lines. Bazak et al. (Bazak et al. 2013) and Yuan et al. (Yuan et al. 2013) have targeted Fe_3_O_4_@TiO_2_ to the nucleus of nasal and cervical cancer cell lines, and achieved light-induced genomic DNA degradation. Possible use of Fe_3_O_4_@TiO_2_ for active targeting coupled with controlled induction of cytotoxicity in neoplastic cells render these nanoconstructs a platform worth exploring for management of neuroblastoma. The possibility of using the nanoparticles targeted to cell nucleus is especially interesting when considering use of ^125^I-MIBG-loaded nanoconstructs; as well as the possibility that the metal oxide component of the nanocomposite may increase radiation sensitivity in combination with external beam radiotherapy.

In this study, we investigated whether bare and surface-modified Fe_3_O_4_@TiO_2_ nanocomposites can enhance radiation-induced ROS production in four genetically distinct neuroblastoma cell lines. The SK-N-AS cell line has a mutated NRAS gene, a non-functional p53, and a single copy of MYCN. In contrast, SK-N-DZ cell line harbors a wild-type p53 (Nakamura et al. 2007), but with an amplified MYCN. Both NBL-W/S and NBL-W/N cell lines have about 100 copies of the MYCN gene (Foley et al. 1991); nevertheless, N-myc protein in NBL-W/S cells is tenfold less than in NBL-W/N due to differences in MYCN mRNA stability (Chagnovich and Cohn 1997). The cell lines used in this study are morphologically of the neuroblastic and substrate adherent phenotypes. Primary neuroblastoma is a heterogonous tumor and generally harbors cells of both phenotypes (Kim et al. 2004). We chose to work with a diverse set of neuroblastoma cell lines in order to observe relative differences in treatment efficiency of nanocomposites under investigation, reflecting the genetic and physiological diversity seen in the clinic. Some of the work, however, was conducted only on SK-N-AS cell line because these cells share many critical aspects of refractory neuroblastoma such as non-functional p53 and mutated NRAS gene (Eleveld et al. 2015).

Neuroblastoma cell lines have been used as a model for investigation of dopamine uptake and synthesis (Cooke et al. 2003) and dopaminergic neuroblastoma has been associated with a poor clinical outcome (Nakagawara et al. 1988). Therefore, we explored 3,4-dihydroxyphenylacetic acid (DOPAC) as a possible coating for Fe_3_O_4_@TiO_2_ nanocomposites. We have previously used dopamine and DOPAC to conjugate small molecules to nanocomposite surfaces (Arora et al. 2012; Bazak et al. 2013; Brown et al. 2018; Chen et al. 2014; Paunesku et al. 2003,2007; Yuan et al. 2013); however, in this study DOPAC itself was designated to serve as nanocomposite surface modification as well as anchor for conjugation of MIBG.

## Results

### Modification of nanocomposite surface coating affects size, aggregation, and cellular uptake

We examined how the coating of nanocomposites with DOPAC or DOPAC conjugated to MIBG might affect nanoconjugate physical characteristics considering that the same nanocomposite colloid was used for preparation of the three final nanoconstructs. Measurements of zeta potentials of these nanoconstructs are shown in Additional file 1: Table S1. It should be noted that MIBG was conjugated to Fe_3_O_4_@TiO_2_ nanocomposites via a DOPAC linker through a reaction using 1-ethyl-3-(3-dimethylaminopropyl) carbodiimide hydrochloride (EDC) and with MIBG as the molecule in higher excess. Under these conditions, no free COOH groups of DOPAC remained on the nanoconjugate surface. We refer to those nanoconjugates as MIBG-coated although it is DOPAC that provides a covalent link between nanoconstruct surface and MIBG (see Additional file 1: Figures S1 and S2). These nanocomposites were imaged by energy dispersive spectroscopy (EDS)–scanning transmission electron microscopy (STEM) (Additional file 1: Figure S1), and elemental maps were generated. There was a clear overlap between iodine (I), iron (Fe), and titanium (Ti) in the elemental map images seen in Additional file 1: Figure S1b, indicating the successful binding of MIBG to the surface of the nanocomposite. Progression through chemical changes on the surface of the nanocomposites correlated with nanoparticle coating was also confirmed by infrared spectroscopy (Additional file 1: Figure S2).

**Fig. 1:**
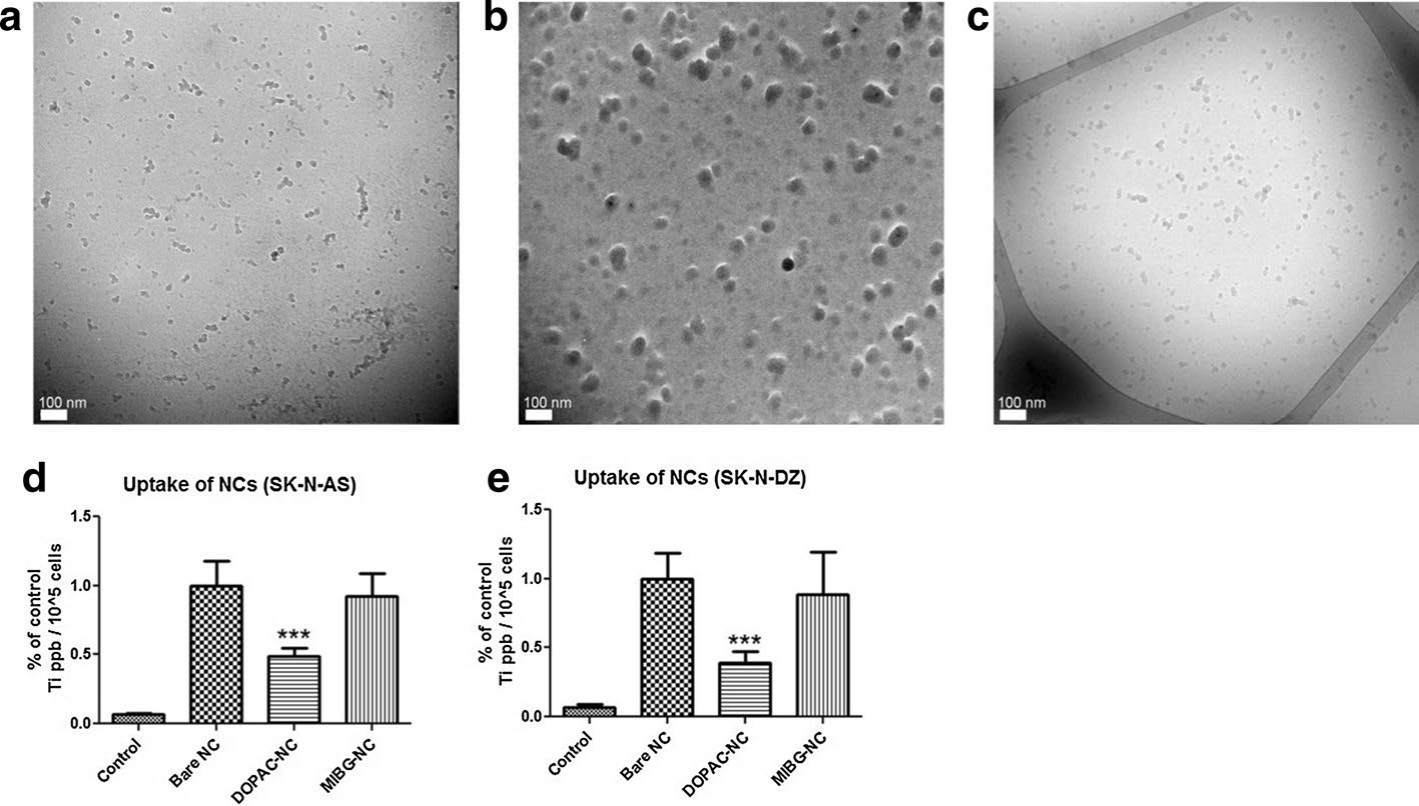
Nanocomposite and nanoconjugate Cryo-TEM show different degrees of aggregation resulting in corresponding differences in nanocomposite uptake by neuroblastoma cells. **a** Cryo-TEM images of bare Fe_3_O_4_@TiO_2_ nanocomposites; **b** DOPAC–Fe_3_O_4_@TiO_2_ nanoconjugates, and **c** MIBG–DOPAC–Fe_3_O_4_@TiO_2_ nanoconjugates mixed with complete cell media, plunge-frozen on lacy carbon grids and imaged under cryogenic conditions at 120 kV. For EDS–STEM and IR spectroscopy of nanocomposites, see supplemental Figs. 1 and 2. **d** SK-N-AS cells were treated with 250 nM bare Fe_3_O_4_@TiO_2_ nanocomposites, DOPAC–Fe_3_O_4_@TiO_2_ nanoconjugates or MIBG–DOPAC–Fe_3_O_4_@TiO_2_ nanoconjugates for 1 h (n = 3), washed and collected after trypsinization. Bar graph shows relative quantity of Ti per 10^5^ cells; **e** same work was done with SK-N-DZ cells. The total concentration of Ti (ppb) per sample was evaluated by ICP-MS and adjusted for the number of cells counted prior to sample processing for ICP-MS. The final concentration of Ti per 10^5^ cells is expressed as a percentage of bare Fe_3_O_4_@TiO_2_ nanocomposite uptake; control value corresponds to Ti background from cells not treated with nanoconstructs. Data presented are an average of at least two independent experiments, each with three biological replicates. Error bars indicate mean ± SD. *** < 0.001 significance level

Bare, DOPAC and MIBG-coated nanoconjugates were produced from the same initial nanocomposites, cleaned by dialysis and suspended in serum-containing cell growth medium. Nanoconjugate suspensions were drop cast onto lacey-carbon grids for transmission electron microscopy (TEM), plunge-frozen, and imaged under cryogenic conditions (Fig. 1a–c). We observed a difference in size and aggregation properties of the different nanoconstructs under these conditions. In complete medium, aggregates formed from bare nanocomposites were much smaller than aggregates of DOPAC-coated nanoconjugates. MIBG-coated nanoconjugates were similar in size and aggregation to the bare nanocomposites. It is possible that the highly polar surface of DOPAC-coated nanoconjugates, with numerous electronegative carboxyl groups exposed to the medium, led to cooperative binding to electropositive components in the complete medium and aggregation of nanoconjugates, while bare and MIBG-coated nanoconstructs acquired only a single layer of protein corona.

We examined cellular uptake of bare, DOPAC- and MIBG-coated nanoconstructs by inductively coupled plasma-mass spectrometry (ICP-MS) (Fig. 1d, e). When neuroblastoma cells (SK-N-AS and SK-N-DZ) were treated for 1 h with equivalent concentrations of Fe_3_O_4_@TiO_2_ nanocomposites (250 nM), there was significantly less uptake of DOPAC-coated nanoconjugates when compared to either bare nanocomposites or MIBG-coated nanoconjugates in both cell lines. This could be explained by the observed difference in aggregation properties of the nanoconstructs. The observed size of bare and MIBG-coated nanoparticles aggregates imaged by TEM was much smaller compared to aggregates of DOPAC-coated nanoconjugates.

### Modification of nanocomposite surface coating modulates subcellular localization of nanoconstructs

**Fig. 2:**
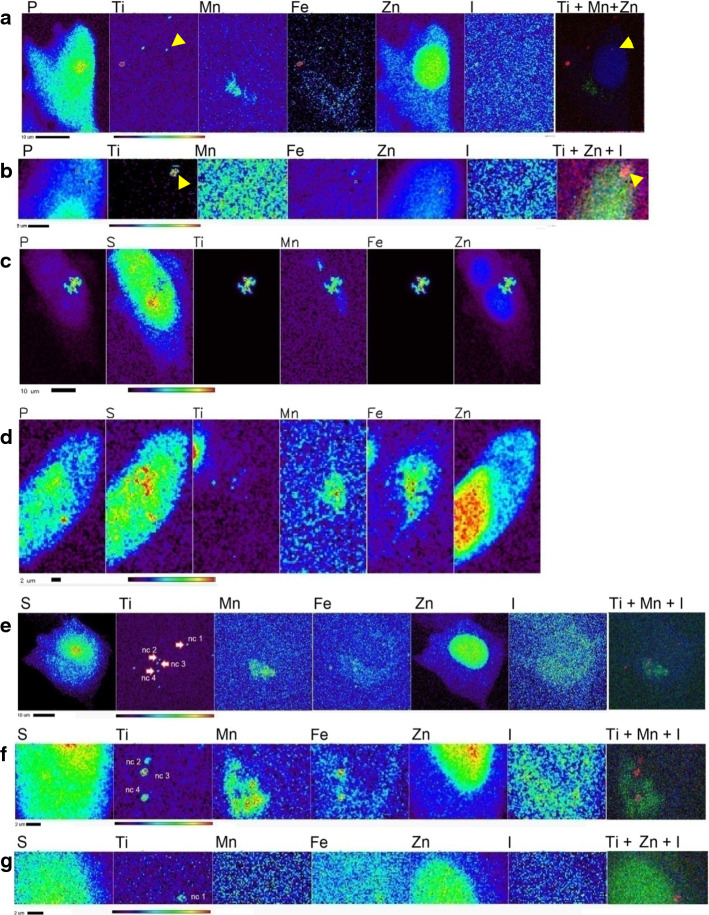
Cryo-XFM imaging of SK-N-AS cells treated with three different nanocomposites. **a** SK-N-AS cells were treated with 250 nM Fe_3_O_4_@TiO_2_ nanocomposites for 1 h. The distribution of Ti and Fe (as proxy for nanocomposites) in SK-N-AS cells was cytoplasmic or associated with the membrane; **b** the same cell was imaged after a 45 degree rotation, with all Ti signal separated from the Zn-rich area of the nucleus; **c** SK-N-AS cells treated with DOPAC–Fe_3_O_4_@TiO_2_ nanoconjugates showing large aggregates with dense concentration of Ti and Fe; **d** SK-N-AS cells also display punctate pattern of smaller aggregates of nanocomposites apparently co-localizing with Mn and Zn signal. **e** SK-N-AS cells treated with MIBG–Fe_3_O_4_@TiO_2_ nanoconjugates. Some co-localization of Ti and Fe puncta with Mn and Zn is observed, indicating either potential mitochondrial or nuclear distribution of MIBG–Fe_3_O_4_@TiO_2_ nanoconjugates. **f** After a + 60 degree rotation, image of the lower portion of same cell is still indicating Ti and Mn co-localization for aggregates nc 3 and nc 4; **g** scan of the upper portion of the same cell after a -60 degree rotation suggests that aggregate nc1 is in fact immediately above the nucleus. Scale bar and elemental concentration indicator (black—no signal to red—highest signal) are located under each image

Using Cryo-X-ray fluorescence microscopy (Cryo-XFM), we examined whether bare, DOPAC- and MIBG-coated nanoconstructs are transported to different subcellular locations after uptake. XFM provides complete elemental spectra for each pixel of the raster-scanned sample and elemental distribution of biologically ubiquitous elements permits identification of cells and subcellular organelles. In figures of XFM maps, sulfur (S) outlines the area of the whole cells because S is a common element in all cellular proteins due to the presence of methionine and cysteine amino acids (Ortega et al. 2004). Zinc (Zn) is present throughout the cell as well, but it is especially abundant in the nucleus (Finney et al. 2007; Glesne et al. 2006; Paunesku et al. 2003) , while the presence of manganese (Mn) indicates locations of mitochondria primarily because of the concentrated presence of manganese superoxide dismutase (MnSOD) (Paunesku et al. 2003,2007). Iodine (I) background in cells is extremely low; this permitted us to use I as a proxy for MIBG distribution; cells left untreated or treated with nanoconjugates without MIBG only showed background I signal. 10-keV incident X-rays excited fluorescence of L shell electrons of I, while all other elements included in Fig. 2 were identified through their K shell fluorescence signals. There is no titanium (Ti) in the cells and the presence of Ti signal indicates location of nanoparticle aggregates (Paunesku et al. 2003,2007). Especially, co-localization of characteristic K alpha electron shell signals of Ti and Fe indicates the position of the metal oxide component of nanoconjugates–Fe_3_O_4_@TiO_2_ nanocomposites themselves. XFM allows quantitative, tomographic, elemental mapping of whole, non-sectioned cells and therefore enables precise measurement and mapping of nanocomposite/nanoconjugate distribution in cells. Moreover, since X-ray fluorescence is an inherent elemental property, this mode of imaging allows for detection of nanoconjugates/nanocomposites without necessitating the addition of dyes to the particle for detection, which could significantly alter the functional properties of that nanoparticle (Grätzel 2004; Rajh et al. 2002). In addition, images obtained with cryogenic XFM technique show elemental content of the cells without elemental re-distribution that can be caused by fixation or drying. Using Cryo-XFM, we observed that bare nanocomposites (Fig. 2a) as indicated by Ti and Fe show a punctate cytoplasmic distribution pattern in SK-N-AS cells. Because one of the nanoparticle aggregates (yellow arrowheads in Fig. 2a) appeared as overlapping with the Zn-high region corresponding to cell nucleus, we have re-imaged this area of the sample after a 45 degree rotation which gave us a different vantage point. This XFM scan showed separation between the cell nucleus and nanocomposite aggregates (yellow arrowheads in Fig. 2b). In Fig. 2c, d, we examined the pattern of distribution of DOPAC-coated nanocomposites in AS cells. Two distinct modes of distribution were observed for cells treated with DOPAC-nanoconjugates. The first, presented in Fig. 2c, was a large aggregation of nanocomposites while the second, in Fig. 2d, was a punctate distribution of smaller nanocomposite aggregates in addition to a large aggregate associated with its perimeter. Cells treated with MIBG-coated nanoconjugates showed only a punctate distribution pattern for Ti and Fe (Fig. 2e–g); these small puncta were associated with I signal as well. Interestingly, several MIBG–Fe_3_O_4_@TiO_2_ aggregates co-localized with Mn, a marker for mitochondria. In order to investigate this from an additional vantage point, we re-scanned areas containing nanoconjugate puncta after ± 60-degree rotations. Elemental co-localization between Ti and Mn was still observed for several of the aggregates (Fig. 2f); at the same time, an apparently nucleus-associated aggregate nc1 was localized in the perinuclear region, rather than within the nucleus itself (Fig. 2g).

Previous reports showed that distribution of MIBG molecule is cytoplasmic and mitochondrial in neuroblastoma cells (Gaze et al. 1991). The study that established this finding implemented electron spectroscopic imaging of thin sectioned cells. However, elemental sensitivity in this work was limited and some have even hypothesized that MIBG diffusion throughout the cell and its presence in specific subcellular compartments may be an artifact of fixation (Clerc et al. 1993). In an effort to prevent MIBG redistribution and obtain information about cells that were not sectioned, we used Cryo-XFM to image iodine distribution in MIBG treated whole frozen hydrated cells. Cryogenic conditions were found to preserve cellular architecture as well as the distribution of small molecules in cultured cells, unlike chemical fixation (Chen et al. 2014).

SK-N-AS and SK-N-DZ cells were grown on silica nitride windows, treated with MIBG, frozen and imaged by Cryo-XFM (Additional file 1: Figure S3a–c). Iodine was distributed through the cytoplasm and perinuclear areas, with very little iodine signal overlapping with the nucleus (Zn rich area). Contrary to previous studies, we demonstrated that MIBG is not localized only to the mitochondria. This finding is confirmed by high-resolution scans of the mitochondria in SK-N-AS (Additional file 1: Figure S3c) and SK-N-DZ (Additional file 1: Figure S3d) cells. Additional file 1: Figure S3e presents a table of the average iodine concentration ratios in the cytoplasm vs. nucleus and the mitochondria vs. cytoplasm. It is important to note that whole-cell XFM imaging without tomographic mapping has to be interpreted with care. For example, the iodine in the cytoplasmic layer directly above the nucleus could be erroneously interpreted as nucleus-associated signal when whole-cell imaging is performed only in 2D. Similar patterns of MIBG distribution were observed in cells treated with lower concentrations of this molecule (Additional file 1: Figure S4).

**Fig. 3:**
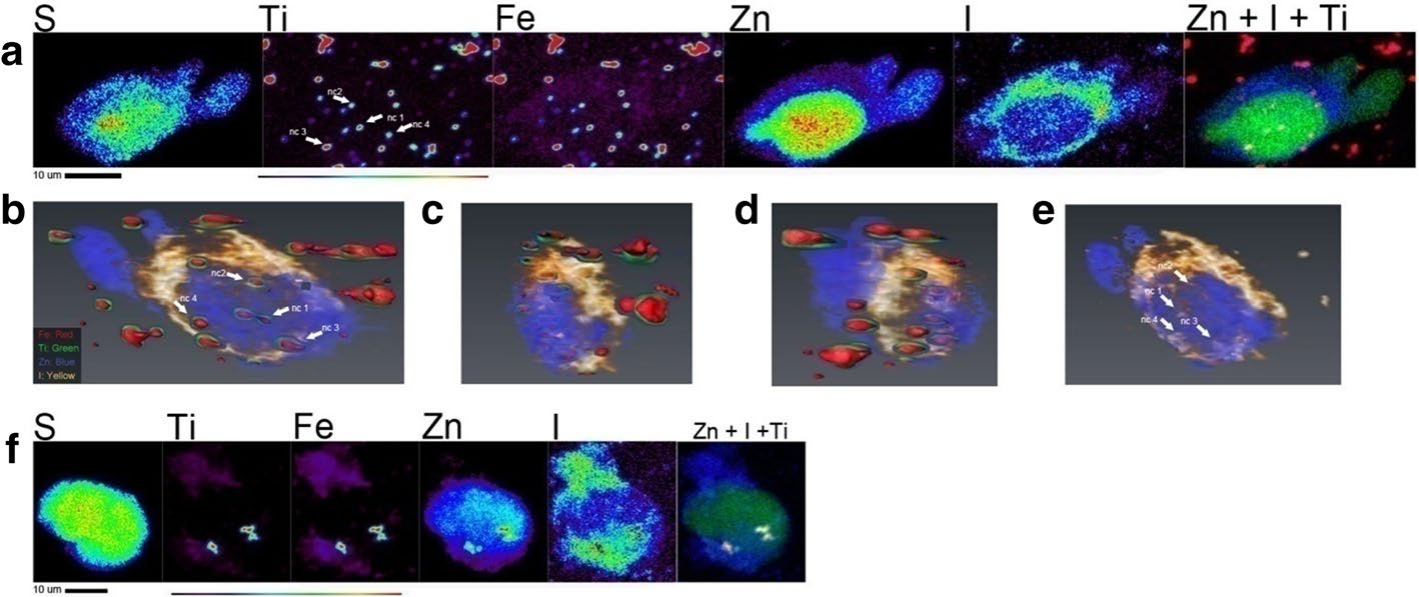
Tomographic imaging of targeted MIBG–Fe3O4@TiO2–B-loop nanoconjugates by Cryo-XFM indicates partial nuclear localization of the nanoconjugates. **a** A single XFM projection of a SK-N-AS cell treated with a 60 µM equivalent treatment of MIBG–Fe3O4@TiO2–B-loop nanoconjugates indicates extensive cytoplasmic and nuclear accumulation of nanoconjugates. Scale bar and elemental concentration indicator (black—no signal to red—highest signal) are located under image. Co-localization image: blue: iodine, red: Ti, green: zinc (overlapping color for all three elements is white). **b**–**d **Screen captures of different single-angle projections of the tomographic reconstruction of the cell seen in **a**. Different rotation projections confirm the localization of Ti and Fe in the nucleus. **e** Rotation projection focused on the iodine signal (indication of MIBG) corresponds to the same locations in the cell nucleus seen in **b**. **f** SK-N-DZ cell treated with MIBG–Fe3O4@TiO2–B-loop nanoconjugates, also suggesting nuclear localization of I and Ti

Neither free MIBG nor MIBG-coated nanoconjugates were observed in neuroblastoma cell nuclei as shown in Fig. 2 and Additional file 1: Figure S3. One motivation for this work was to develop an approach to deliver MIBG into cell nucleus, with the view to enable delivery of ^125^I immediately adjacent to the genomic DNA. In an effort to determine if it is ever possible for MIBG–Fe_3_O_4_@TiO_2_ nanocomposites to reach the nucleus, we have added onto nanocomposite surface a targeting moiety - an EGF-mimicking “B-loop” peptide (Yuan et al. 2013). We have previously shown that Fe_3_O_4_@TiO_2_ nanocomposites conjugated to this peptide bind to epidermal growth factor receptor (EGFR) and utilize the EGFR cellular trafficking to localize partially to the cell nucleus of HeLa cells (Yuan et al. 2013). MIBG–Fe_3_O_4_@TiO_2_–EGFB-loop nanoconjugates were used to treat cells grown on silica nitride windows, at a concentration equivalent to 60 µM MIBG and 637 nM nanocomposites. Treatment lasted for 90 min in serum-free medium at 37 °C; windows were washed, plunge-frozen in liquid ethane and imaged with the Bionanoprobe X-ray fluorescence imaging instrument at APS. In Fig. 3a, elemental maps of an SK-N-AS cell treated with MIBG–Fe_3_O_4_@TiO_2_-B-loop nanoconjugates are presented. The nanoconjugates were detected within the nucleus, although the majority of nanoconjugates remained in the cytoplasm. To confirm this finding, a full tomographic dataset of 2D maps covering a total rotation over 138 degrees was obtained. Image reconstruction and visualization at a variety of angles are shown in Fig. 3b–e and Additional file 2: Video. Tomography confirmed nuclear localization of MIBG–Fe_3_O_4_@TiO_2_-B-loop nanoconjugates as indicated by the presence of Ti, Fe as well as I. Elemental quantification of Ti in a region of interest drawn for the nucleus accounted for 35.3% of complete Ti inside the cell, similar to our findings with nanocomposites coated with B-loop in the HeLa cell line (Yuan et al. 2013). The trafficking of EGFR to the nucleus is partial (Dittmann et al. 2010; Dittmann et al. 2008; Lin et al. 2001 11533659; Lo et al. 2006; Wang et al. 2010; Wang et al. 2010) and only a portion of the targeted nanoconjugates can be expected to reach the nucleus. Figure 3f presents image of an SK-N-DZ cell from the cell sample treated with MIBG–Fe_3_O_4_@TiO_2_-B-loop nanoconjugates at the same concentration as the SK-M-AS cell line. A full tomographic dataset for this cell was not obtained due to time constraints for tomographic XFM imaging; however, a strong overlap between the Zn signal (nucleus) and Ti signal (nanoconjugate) suggests that the nanoconjugate aggregate is spatially associated with the nucleus. Although it has been suggested that nuclear pores uptake can accommodate structures from 39 to 234 nm (Misra and Sahoo 2010; Pante and Kann 2002; Paulo et al. 2011), larger aggregates that cannot completely translocate into the nucleus may nevertheless remain lodged in the nuclear membrane.

### Nanoconjugate surface coating influences cell viability in nanoconjugate-treated neuroblastoma cells

Because cell treatments with metal oxide nanoparticles in general are never free of side-effects, we wished to see whether such effects could be used to additional determent of cancer cells. We examined the effect of nanoconjugates on viability of neuroblastoma cell lines. Treatment with bare Fe_3_O_4_@TiO_2_ nanocomposites at concentrations up to 250 nM did not significantly affect viability of either SK-N-AS (Additional file 1: Figure S5a), SK-N-DZ (Additional file 1: Figure S5b) or NBL-W/S (Additional file 1: Figure S5c) cell lines measured by MTS assay 72 h after nanoconstructs treatment. However, viability of NBL-W/N cells (Additional file 1: Figure S5d) treated with 250 nM bare nanocomposites was reduced to nearly 50%. MIBG molecule concentrations of up to 30 μM had minimal effect on viability of SK-N-AS and SK-N-DZ cells (Additional file 1: Figure S5e, f).

**Fig. 4:**
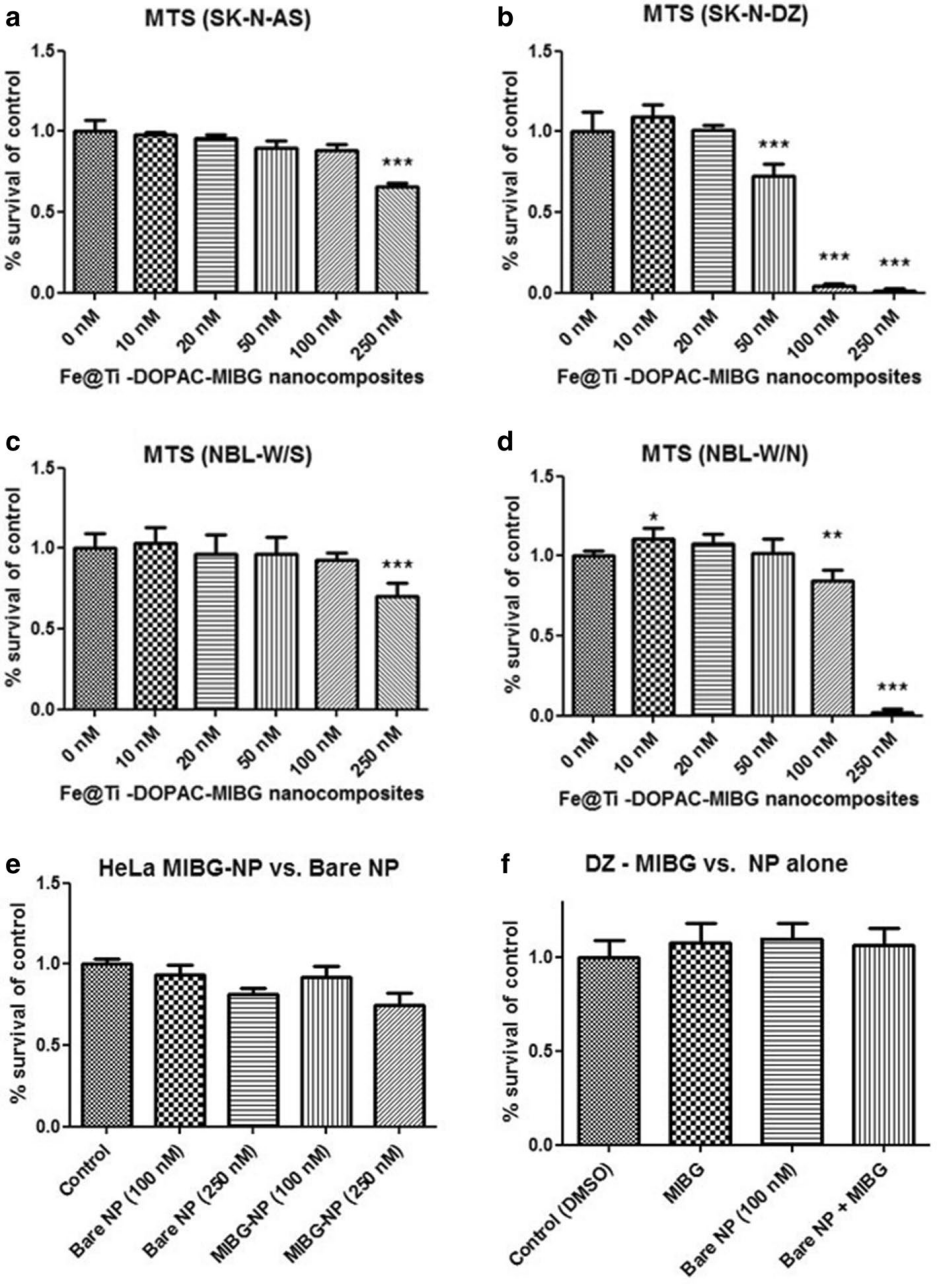
Effect of MIBG nanoconjugates on neuroblastoma cell viability. **a** SK-N-AS cells; **b** SK-N-DZ; **c** NBL-W/S and **d** NBL-W/N were treated with varying concentrations of Fe_3_O_4_@TiO_2_–MIBG nanoconjugates. Loss of viability was observed by MTS assay as detailed. **e** HeLa cells treated with 100 nM or 250 nM Fe_3_O_4_@TiO_2_ bare nanocomposites or MIBG nanoconjugates. **f** SK-N-DZ cells treated with free MIBG (9.3 µM), bare nanocomposites (100 nM), or bare nanocomposites + free MIBG. No decrease in cell viability was found in either treatment condition *: < 0.05 significance level, **: < 0.01 significance level, *** < 0.001 significance level. Datapoints presented are an average of 5 biological replicates. Error bars indicate mean ± SD

Interestingly, all four cell types have shown a statistically significant decrease in viability when treated with MIBG–Fe_3_O_4_@TiO_2_ nanoconjugates (Fig. 4a–d). Maximal nanocomposite concentration tested was 250 nM while corresponding maximal concentration of MIBG was 23.59 μM. Viability of SK-N-AS and NBL-W/S cells was affected only at the 250 nM concentration of MIBG–Fe_3_O_4_@TiO_2_ nanoconjugates, while the loss of viability of SK-N-DZ cells was significant starting at 50 nM MIBG nanoconjugate concentration and at 100 nM concentration in cell line NBL-W/N. We also investigated if this effect on cell viability was notable only in neuroblastoma cells by investigating MIBG–Fe_3_O_4_@TiO_2_ nanoconjugate effect on a non-neuroblastoma cell line, HeLa (Fig. 4e). These cells do not express a norepinephrine receptor critical for MIBG-specific uptake (Glowniak et al. 1993). We found that there was no significant difference in cytotoxicity between HeLa cells treated with bare nanocomposites or MIBG nanoconjugates. Finally, we examined if the effect of MIBG nanoconjugates on cytotoxicity could be explained by a process whereby free MIBG could induce additional nanocomposite uptake or another cellular response, leading to the observed significant effect on cytotoxicity. When a co-treatment of the most responsive of the four cell lines SK-N-DZ cells was done with simultaneous addition of bare nanocomposites (100 nM) and free MIBG (in concentration of 9.3 μM, equivalent to what was present on 100 nM MIBG-coated nanoconjugates concentration), no significant decrease in viability was observed (Fig. 4f).

### Decrease of cell viability caused by ionizing radiation is enhanced in the presence of DOPAC–Fe_3_O_4_@TiO_2_ nanoconjugates

**Fig. 5:**
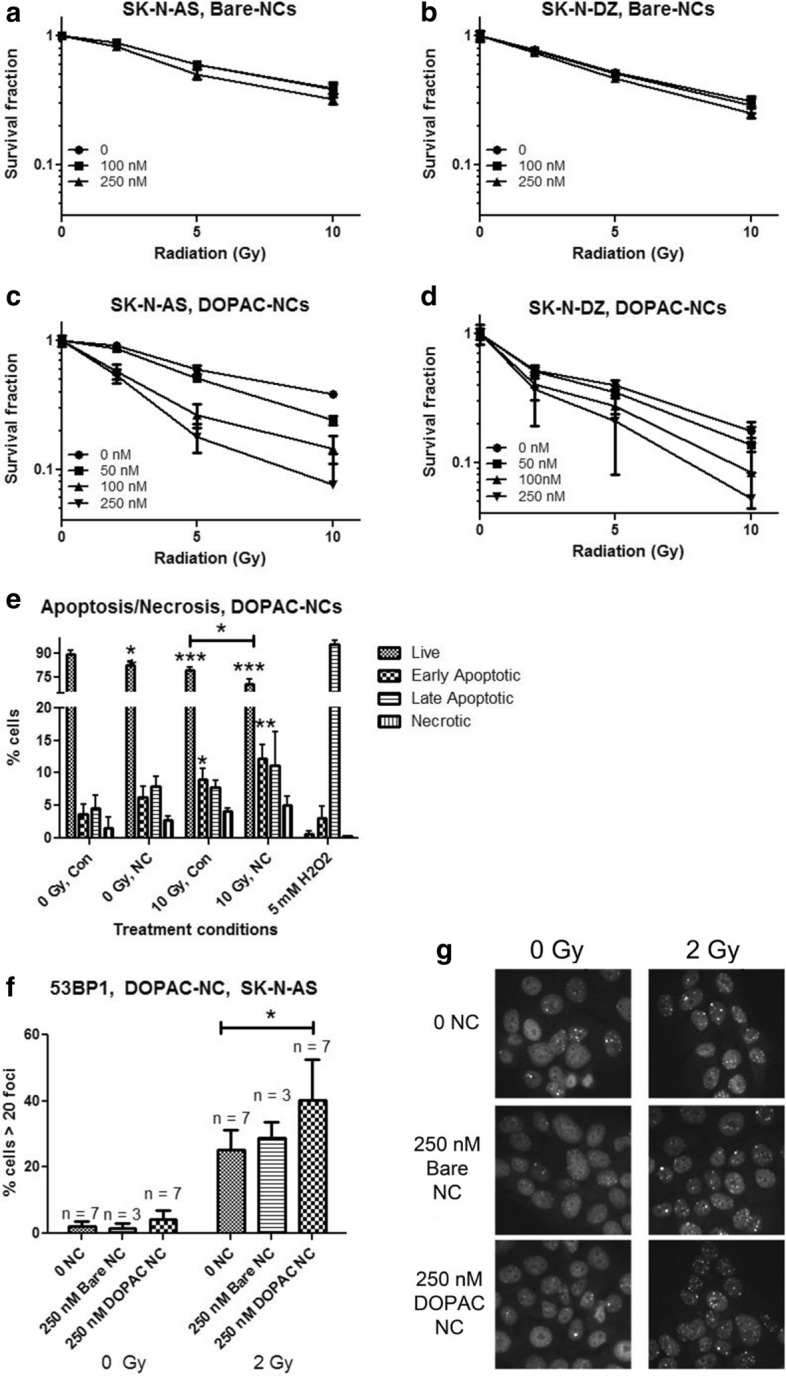
Radiosensitizing effects of bare nanocomposites and DOPAC-coated nanoconjugates. **a**, **c** SK-N-AS and **b**, **d** SK-N-DZ cells were irradiated in the presence of bare (Bare-NCs) or DOPAC-coated (DOPAC-NCs) nanoconstructs of different concentrations. Curves were generated by adjusting cell viabilities to 100% for non-irradiated cells in each nanoconstruct treated group. A statistically significant radiosensitizing effect was observed at 250 nM bare nanocomposites in both cell lines, particularly at 10 Gy. Datapoints presented are average of 5 biological replicates and are representative of at least two independent MTS experiments. Error bars indicate mean ± SD. **e** Annexin V/propidium iodide flow cytometry assay of SK-N-AS cells 24 h after irradiation (0 or 10 Gy) preceded by treatment with 250 nM DOPAC-nanocomposites. H_2_O_2_ was the positive control. Three independent experiments were done, with 3 biological replicates per experiment. Con = cells not exposed to nanoconjugates; NC = DOPAC nanoconjugate treatment; **f** percentage of SK-N-AS cells with > 20 foci per nucleus, for untreated or treated with 250 nM bare nanocomposites or 250 nM DOPAC-nanoconjugates for one hour and irradiated as indicated. 53BP1 foci were stained by immunocytochemistry while the nuclei were counterstained with propidium iodide (PI). At least 100 cells were counted for each treatment group per replicate. N = total number of biological replicates from 4 independent experiments of 1–2 replicates each. There was a significant increase in the percentage of cells with > 20 foci after 2 Gy treatment. **g** Representative images of cells shown in **f**. Error bars indicate mean ± SD. * < 0.05 significance level, ** < 0.01 significance level, *** < 0.001 significance level when treatment sample is compared to untreated and/or unirradiated control

Neuroblastoma cell lines used in this study differed significantly with respect to radiation sensitivity (Additional file 1: Figure S6), with cell line SK-N-AS being the most radiation resistant. We evaluated the use of bare nanocomposites, free DOPAC and DOPAC-coated Fe_3_O_4_@TiO_2_ nanoconjugates as possible radiation sensitizers (Fig. 5, Additional file 1: Figure S7). Typically, a sensitizer enhancement ratio (SER) is calculated as the ratio of the dose necessary to achieve a particular level of cell killing in the absence of the sensitizer and in the presence of the sensitizer (Tubiana 2005). In this case, we define the nanocomposite radiation enhancement ratio (NRER) to be the ratio of the percentage of cell viability at a particular radiation dose in the absence of nanocomposites to the percentage of viability in the presence of nanocomposites. An overview of cell viability is presented in Tables 1 and 2. Significant enhancement of radiation effects was observed in SK-N-AS cells for exposures to 2, 5, and 10 Gy in the presence of 250 nM bare nanocomposites. In SK-N-DZ cells, significant enhancement was observed at 5 and 10 Gy in the presence of 250 nM bare nanocomposites. The calculated NRER for bare nanocomposites at 10 Gy was 1.2 for SK-N-AS cells and 1.24 for SK-N-DZ cells.

**Table 1:** Cell viabilities in the presence of bare nanocomposites following irradiation

Fe_3_O_4_@TiO_2_		0 nM	100 nM	250 nM
SK-N-AS	0 Gy	100 ± 2.22	100 ± 2.00	100 ± 1.42
	2 Gy	88.15 ± 2.85	88.10 ± 3.60	82.22 ± 2.50**
	5 Gy	60.00 ± 1.63	59.61 ± 1.73	50.30 ± 4.49**
	10 Gy	38.90 ± 3.16	38.40 ± 4.53	32.35 ± 2.49**
SK-N-DZ	0 Gy	100 ± 7.42	100 ± 9.29	100 ± 1.86
	2 Gy	78.68 ± 2.70	77.00 ± 3.18	74.55 ± 3.18
	5 Gy	52.13 ± 2.46	51.35 ± 2.47	46.89 ± 1.64**
	10 Gy	31.14 ± 2.78	29.01 ± 3.53	25.03 ± 2.05**

Table of cell viabilities (expressed as % of non-irradiated treatment control for a given concentration of nanoconjugates, after adjustment for baseline cytotoxicity) for SK-N-AS and SK-N-DZ cells treated with varying concentrations of bare nanocomposites and different doses of ionizing radiation (Fig. 5). Entries contain mean ± SD. **: < 0.01 significance level, *** < 0.001 significance level.

**Table 2:** Cell viabilities in the presence of DOPAC–Fe_3_O_4_@TiO_2_ nanoconjugates following irradiation

DOPAC–Fe_3_O_4_@TiO_2_		0 nM	100 nM	250 nM
SK-N-AS	0 Gy	100 ± 5.06	100 ± 4.50	100 ± 10.11
	2 Gy	91.84 ± 5.50	57.60 ± 7.28***	53.50 ± 6.60***
	5 Gy	59.38 ± 4.79	26.40 ± 5.56***	17.94 ± 4.54***
	10 Gy	38.11 ± 2.35	14.53 ± 3.50***	7.58 ± 5.67***
SK-N-DZ	0 Gy	100 ± 10.12	100 ± 9.28	100 ± 17.87
	2 Gy	51.97 ± 4.34	40.35 ± 10.15*	36.83 ± 17.71
	5 Gy	40.06 ± 3.35	27.55 ± 3.86***	20.78 ± 12.76**
	10 Gy	17.52 ± 3.23	8.25 ± 3.84**	5.20 ± 10.43*

Table of cell viabilities (expressed as % of non-irradiated treatment control for a given concentration of nanoconjugates, after adjustment for baseline cytotoxicity) for SK-N-AS and SK-N-DZ cells treated with varying concentrations of DOPAC-nanocomposites combined with varying doses of ionizing radiation, from Fig. 5. Entries contain mean ± SD. *: < 0.05 significance level, **: < 0.01 significance level, *** < 0.001 significance level

As detailed above, we noted changes in uptake, subcellular localization, and size of aggregates of DOPAC–Fe_3_O_4_@TiO_2_ nanoconjugates compared to bare nanocomposites (Figs. 1 and 2). Neither bare nor DOPAC-covered nanoconjugates migrated to cell nucleus and we did not anticipate that they would show significantly different radiosensitization properties; nevertheless, NRER of DOPAC-covered nanoconjugates was markedly higher. In SK-N-AS and SK-N-DZ cells, significant radiation sensitization was observed at 2, 5, and 10 Gy (Table 2) in combination with 100 and 250 nM nanoparticle concentrations. In this case, the NRER calculated for 250 nM DOPAC-nanoconjugates at 10 Gy was 5.03 and 3.37 for SK-N-AS and SK-N-DZ cells, respectively. To examine if this effect could be due to DOPAC alone, we repeated irradiations of neuroblastoma cells in the presence of DOPAC (Additional file 1: Figure S7). Treatment of SK-N-AS and SK-N-DZ cells with concentrations of DOPAC equivalent to the DOPAC concentration bound to the nanocomposite surface (13 μM, 33 μM), or even at significantly higher concentrations (50 μM, 100 μM), had no additive or synergistic effect with irradiation in either cell line.

We then examined the mode of cell death in SK-N-AS cells, the most radio-resistant cell line in this study (Fig. 5e). SK-N-AS cells were exposed to 10 Gy of ionizing radiation alone, 250 nM DOPAC-nanoconjugates alone, or a combination of the two. Cells were then incubated for 24 h, stained with Annexin-FITC and propidium iodide (PI), and evaluated by flow cytometry. Apoptosis was apparent in all treated samples. There was also a significant decrease in the number of live cells in samples treated with a combination of 10 Gy and DOPAC-nanoconjugates, compared to cells exposed either to 10 Gy alone or nanoconjugate alone. The percentage of live cells in 10 Gy and DOPAC-nanoconjugates treated cells was 70.43% at this time point, compared to 79.03% live cells after exposure to 10 Gy alone or 82.55% live cells after treatment with DOPAC–Fe_3_O_4_@TiO_2_ alone. There was also a significant increase in early apoptotic cells in samples exposed to 10 Gy and DOPAC-nanoconjugates (12.26%) compared to untreated control (3.7%).

Because intracellular distribution of DOPAC-nanoconjugates was cytoplasmic and the most likely physical product of interaction between the nanoparticles and ionizing radiation is the production or reactive oxygen species (ROS), we considered it questionable whether ROS produced by the nanoconjugates would reach genomic DNA to increase the damage caused to it by ionizing radiation. DNA damage is considered to be the most important effect of irradiation and the primary cause of radiation-induced cell death. To investigate the extent of double-stranded DNA breaks in the presence of DOPAC–Fe_3_O_4_@TiO_2_ nanoconjugates, we performed immunocytochemistry for p53-Binding Protein 1 (53BP1) foci, indicating sites of DNA double-strand breaks (Fig. 5f, g). SK-N-AS cells were treated with 2 Gy radiation alone, 250 nM bare nanocomposites alone, 250 nM DOPAC–Fe_3_O_4_@TiO_2_ nanoconjugates alone, or a combination of 2 Gy and either nanoconstruct. One hour after nanoconstruct treatment, cells were irradiated and allowed to recover in the incubator for 4 h. Then the cells were fixed and stained for 53BP1 foci and the nuclei were counterstained with propidium iodide (PI). Foci were counted for at least 100 cells per treatment group and the percentage of cells with 20 or more foci was determined. At 0 Gy, as expected, the cells with 20 or more foci were few. At 2 Gy there was a notable increase in the number of foci in all samples, but the greatest increase was seen in irradiated cells pretreated with DOPAC-nanoconjugates. These results indicate that the cellular uptake of DOPAC-nanocomposites leads to an increased accumulation of DNA double-strand breaks following irradiation with a dose as small as 2 Gy. It is worth noting that this dose of gamma irradiation did not significantly decrease the viability of either non-treated SK-N-AS cells or cells treated with bare nanocomposites (Table 1, Fig. 4a). However, the same experiments have shown that a 2 Gy exposure of cells previously treated with 250 nM DOPAC-nanoconjugates has led to a 47% decrease in cell viability (Table 2, Fig. 4c). Despite the correlation between the treatment with DOPAC-nanoconjugates, increase in DNA damage caused by ionizing radiation and increase in cell death, we cannot necessarily claim that accumulation of DNA damage or cell death were directly related to ROS production by DOPAC-nanoconjugates. Alternative explanations for these findings are possible, for example, perturbation of the cell membrane may lead to changes in signaling cascades that result in decreased DNA repair capacity. Our DNA damage evaluation was done at a late timepoint when much of the initial DNA damage in the cell may have already been repaired. Additional research will need to be done to establish the precise role of DOPAC-nanoconjugates in the increase of the genomic DNA damage accumulation after irradiation.

## Discussion

Neuroblastoma is a highly lethal pediatric cancer where nearly 50% of patients present with metastasis at the time of diagnosis (DuBois et al. 1999; Maris et al. 2007). Recently, it has become evident that in patients who experience relapse, the recurrent tumor cells exhibit little resemblance to the original tumor genotype (Eleveld et al. 2015; Schramm et al. 2015). New mutations in genes associated with RAS–MAPK pathway accumulate in the course of disease development contributing to a poor prognosis (Eleveld et al. 2015; Schramm et al. 2015). Thus, many treatments that were initially successful become ineffective when relapse occurs (Huang and Weiss 2013). Management of relapse requires aggressive therapies including radionuclide treatments that are often limited because of associated toxicities. Several recent clinical studies used either high-dose or peptide-modified MIBG-based therapies (Kayano et al. 2020; Sugiyama et al. 2020). Despite some promising findings of these studies, development of more efficient treatments that work on most neuroblastoma phenotypes and for refractory disease is critically important. Few recent studies using different nanotechnology platforms to target neuroblastomas suggest that bionanotechnology is a promising direction for research in neuroblastoma (Alfei et al. 2020; Di Paolo et al. 2020).

In an effort to exploit nanotechnology to enhance currently available treatment modalities, this study has examined a core–shell Fe_3_O_4_@TiO_2_ nanocomposite as a potential radiosensitizer in neuroblastoma cell lines. The effects of bare, MIBG and DOPAC-coated Fe_3_O_4_@TiO_2_ nanocomposites alone or combined with radiation were evaluated in four neuroblastoma cell lines with dissimilar genetic makeup. In general, the cell line SK-N-AS showed the greatest resistance to radiation and both bare and DOPAC-coated Fe_3_O_4_@TiO_2_ nanoconjugates. In contrast, SK-N-DZ and NBL-W/N cell lines were sensitive to nanocomposites on their own as well as radiation alone. Combined use of radiation and nanocomposites led to more cell death than either treatment alone in SK-N-AS and SK-N-DZ cell lines. Interestingly, enhancement of radiation caused loss of cell viability due to the presence of nanoconstructs was similar in both cell lines.

Exploiting the native cellular mechanisms of endocytosis and nuclear translocation of activated EGFR (Dittmann et al. 2010,2008; Lin et al. 2001; Lo et al. 2006; Wang et al. 2010; Wang et al. 2010), we had previously used EGF-mimicking peptide to promote cellular uptake and nuclear translocation of Fe_3_O_4_@TiO_2_ nanoconjugates (Yuan et al. 2013). Neuroblastomas widely express EGFR (Ho et al. 2005; Karmakar et al. 2009; Zage et al. 2013), and we used the same EGF-mimicking peptide to target nanoconstructs into nuclei of neuroblastoma cells as well. Fe_3_O_4_@TiO_2_ nanoconjugates without the EGFR-targeting peptide tend to accumulate in cytoplasm of SK-N-AS and SK-N-DZ neuroblastoma cells (Fig. 2). We anticipated this as we have found cytoplasmic distribution of other non-targeted nanoparticles in other types of cell lines in the past (Arora et al. 2012; Brown et al. 2018; Thurn et al. 2011,2009). In contrast, XFM imaging has demonstrated that EGFR-targeting peptide promoted cellular and nuclear localization of these nanoconjugates (Fig. 3). While EGFR targeting may deliver nanoconjugates to the cells and cell nuclei, other peptides such as G_D2_ disialoganglioside could be used for targeting as well. G_D2_ disialoganglioside is expressed on 98% of neuroblastoma cells, and it is already in use for antibody-based neuroblastoma therapies (Iavarone et al. 1993; Maris et al. 2007).

MIBG has been shown to accumulate in nearly 85–90% of neuroblastoma through their expression of adrenaline/norepinephrine transporters (Carlin et al. 2003). Radioactive ^131^I-MIBG is currently used for diagnosis (Paltiel et al. 1994) as well as the treatment of high-risk and refractory neuroblastoma (Kayano et al. 2020; Matthay et al. 2007; Sugiyama et al. 2020). In this study, we confirmed previous assessment that MIBG may accumulate in mitochondria (Gaze et al. 1991) as well as cytosol (Additional file 1: Figures S3 and S4). MIBG-coated nanoconjugates did appear associated with the same cellular regions as well (Fig. 2). Nevertheless, radiosensitization of cells treated with MIBG nanoconjugates was less than 1.5-fold and further studies will be necessary to explore possible advantages of MIBG-coated nanoparticle preparation. One of the ideas that inspired us to add EGFR-targeting peptide to the MIBG-coated nanoconstructs was to evaluate whether this mode of MIBG delivery into cell nucleus may be successful. In our future work, we hope to explore effects of nucleus-targeted ^125^I-MIBG-loaded nanoconstructs. This short-range emitter would produce less toxicity to normal tissues, and it could be expected to be efficient against micrometastases; in addition, its distribution in the patient could be evaluated by scintigraphy.

## Conclusions

Our studies demonstrated that administration of bare and MIBG-coated Fe_3_O_4_@TiO_2_ nanocomposites led to a modest increase in radiation toxicity in several neuroblastoma cell lines exposed to external beam radiation. On the other hand, DOPAC-coated nanocomposites despite aggregation and low cellular uptake dramatically increased sensitivity to radiation treatment in the neuroblastoma cells tested. Distribution of nanoconstructs and free MIBG in cells was studied by X-ray fluorescence microscopy. All non-targeted nanoconstructs accumulated, as expected, in cytoplasm, and occasionally, in the mitochondria. Nanocomposites targeted through the presence of the an EGF-mimicking peptide, on the other hand, partially entered the cell nuclei. An attractive potential future avenue of research would be to target radiolabeled-MIBG molecules into the cell nucleus using nucleus-targeted nanoconstructs. Such nanoconjugates could act as radiosensitizers for external beam therapy as well as deliver internal emitters close to the genomic DNA.

## Methods

### Nanocomposite synthesis and characterization

Fe_3_O_4_ core and TiO_2_ shell NPs (Fe_3_O_4_@TiO_2_) were synthesized through a modified low-temperature alkaline hydrolysis method as previously described (Arora et al. 2012). Fe_3_O_4_ nanoparticle cores were synthesized by stirring a solution of FeCl_2_ and FeCl_3_ in 24 mM citric acid for 3 h at room temperature. The mixture was then allowed to gel in static air at 70 °C for 24 h, forming the Fe_3_O_4_ core nanoparticles 1.5 to 3 nm in size. This solution was chilled and stirred vigorously with the gradual addition of chilled TiCl_4_ in HCl at 4 °C, allowing for the Ti shell to form. Elemental concentration determination of nanocomposite suspension was performed by measuring titanium and iron concentrations by ICP-MS at the Northwestern University Quantitative Bioelemental Imaging Center on an X series II ICP-MS (Thermo scientific, West Palm Beach, FL). The calculation used to determine the molarity of nanocomposites was previously described (Arora et al. 2012), using atomic force microscopy (AFM) sizing calculations and elemental concentrations determined by ICP-MS.

Following synthesis, Fe_3_O_4_@TiO_2_ nanocomposites were dialyzed (dialysis tubing pore size = 2,000 MWCO) in 10 mM Na_2_HPO_4_ (pH 4.5) and stored at 4 °C. Under these conditions, phosphate molecules attach to the nanoparticle surface; this preparation constituted the “bare” nanocomposites (Michelmore et al. 2000 ).

MIBG (Sigma-Aldrich, St. Louis, MO) was bound with 3,4-dihydroxyphenylacetic acid (DOPAC) through a peptide bond-forming reaction using EDC (Thermo Scientific), following the manufacturer’s instructions. The final concentration of MIBG–DOPAC was 2.34 mM. DOPAC was used as a linker because it has a high affinity for the surface of nano-sized TiO_2_ being a catechol with a carboxyl group that can form a peptide bond with the amino group of MIBG (Creutz and Chou 2008; Paunesku et al. 2007; Thurn et al. 2009). Fe_3_O_4_@TiO_2_ nanocomposites were mixed overnight with DOPAC–MIBG in an oxygen-free atmosphere; the resultant nanoconjugates were dialyzed in 10 mM sodium phosphate buffer (dialysis pore size of 2,000 MWCO). Using a calculation approach previously described (Arora et al. 2012), we estimated that mixing DOPAC–MIBG solution as prepared in equal volume with 22.3 μM Fe_3_O_4_@TiO_2_ nanocomposites leads to DOPAC–MIBG covering roughly 70% of the nanoparticle surface.

DOPAC–Fe_3_O_4_@TiO_2_ nanoconjugates were prepared by combining equal volumes of 7.838 mM DOPAC with 28.93 μM Fe_3_O_4_@TiO_2_ nanocomposites in an oxygen-free environment followed by mixing the conjugation reaction overnight at 4 °C. DOPAC–Fe_3_O_4_@TiO_2_ nanocomposites were then dialyzed for 2 h in 10 mM Na_2_HPO_4_ buffer. We estimated that under these circumstances, molecules of DOPAC covered roughly 100% of the nanoparticle surface.

Elemental makeup and the shape of Fe_3_O_4_@TiO_2_–MIBG nanoconjugates were evaluated by EDS–STEM, which was performed at the Northwestern University’s Atomic and Nanoscale Characterization Experimental Center (Additional file 1: Figure S1a–c). In preparation for EDS–STEM, Fe_3_O_4_@TiO_2_–MIBG nanoconjugates were diluted 1:100 in ddH_2_O, drop cast onto 150 square mesh copper grids with a carbon film support, allowed to dry, and then imaged on a Hitachi HD-2300 Dual EDS Cryo STEM (Additional file 1: Figure S1a).

Infrared determination of nanocomposite coating (Additional file 1: Figure S2) was performed using infrared spectroscopy at the infrared environmental imaging (IRENI) instrument at University of Wisconsin Synchrotron Center. Droplets of different nanoconjugate colloids or component solutions were cast and dried on Ultralene membrane supports and scanned in 2D. Chemograms for the areas of interest were obtained. The addition of each new nanoconjugate coating can be followed by appearance of new spectral features (Additional file 1: Figure S2).

Zeta-potential (ZP) measurements of the nanoconjugates were also obtained (Additional file 1: Table S1) using a protocol that was adapted from the procedure recommended by the Nanotechnology Characterization Laboratory at the National Cancer Institute (Clogston 2009). Bare nanocomposites, DOPAC–Fe_3_O_4_@TiO_2_, and MIBG–Fe_3_O_4_@TiO_2_ nanoconjugates were diluted 1:100 in 10 mM filtered NaCl and ZP calculated using the following constants (temp: 25 °C, viscosity: 0.891, dielectric constant: 78.6, Henry function: 1.5, refractive index: 1.33) on a Zeta sizer Nano (Malvern, Worcestershire, United Kingdom). Fe_3_O_4_@TiO_2_–MIBG nanoconjugates have a mean ZP of -40.887 ± 1.85, bare (phosphate covered) Fe_3_O_4_@TiO_2_ nanoconjugates have a mean ZP of -37.1 ± 1.91, and DOPAC-coated nanocomposites have a mean ZP of -33.367 ± 0.71.

Nanosight measurements were also performed using a Nanosight LM10-HS (Malvern, Worcestershire, United Kingdom) in lieu of dynamic light scattering (Additional file 1: Table S2). Because of the polydispersity of nanocomposites, presence of nanocomposite aggregates and the non-spherical shape of these objects, these results are not as reliable a source of nanoparticle size information as the AFM or TEM data.

To further confirm the spatial and aggregation characteristics of different nanocomposites, Cryo-TEM (Fig. 1a–c) was performed at the Northwestern University Biological Imaging Facility. Nanocomposite 1:100 dilutions in full media (DMEM + 10% FBS) of bare (phosphate coated) Fe_3_O_4_@TiO_2_ nanocomposites, DOPAC–Fe_3_O_4_@TiO_2_ nanoconjugates, and MIBG–Fe_3_O_4_@TiO_2_ nanoconjugates were drop cast on plasma-treated lacey carbon TEM grids, plunge-frozen in liquid ethane using a FEI Vitrobot Mark IV, and Cryo-TEM was performed using a JEOL 1230 TEM at 120 kV. Image brightness levels were adjusted to enhance contrast. The spatial and aggregation properties of different nanocomposites are similar, close to the value obtained by AFM and smaller than the size estimates obtained from the light scattering measurements done with the Nanosight instrument.

EGFR B-loop peptide (DOPAC–MYIEALDKYAC-COOH) and scrambled peptide (DOPAC–EAKLDYMCIYA-COOH) were synthesized by the IBNAM (now The Simpson Querrey Institute for Bionanotechnology) Core Facility of Northwestern University’s Institute for Bionanotechnology in Medicine. The DOPAC group at the N-terminus of the peptide served as a linker to conjugate the peptide to the TiO_2_ surface of the nanoparticles. B-loop peptide was dissolved to a concentration of 700 μM in ddH_2_O, bubbled with N_2_, then mixed in equal volume with 22.3 μM MIBG-nanocomposites or 28.93 μM bare nanocomposites. A separate set of nanocomposites was conjugated to a scrambled peptide. The conjugation was performed in an oxygen-free atmosphere overnight at 4 °C. At a concentration of 350 μM peptide and 11.15 μM MIBG–Fe_3_O_4_@TiO_2_, it was estimated that the B-loop peptide should cover roughly 23% of the MIBG–nanoparticle surface, while with bare nanocomposites the surface coverage was estimated to be roughly 18% (Arora et al. 2012). Experiments with cells in culture were performed within 4 h following conjugation.

### Cell culture

Neuroblastoma cell lines SK-N-AS and SK-N-DZ and cervical cancer cell line HeLa were purchased from American Type Culture Collection (ATCC, Manassas, Virginia). These cells were grown in DMEM supplemented with 10% non-heat inactivated FBS with penicillin/streptomycin and non-essential amino acids at 37 °C and 5% CO_2_. Neuroblastoma cell lines NBL-W/S and NBL-W/N were a generous gift from S.L. Cohn (Department of Pediatrics, University of Chicago, Chicago, IL). These cells were grown in RPMI-1640 medium supplemented with 10% heat-inactivated FBS with penicillin/streptomycin and L-glutamine.

### Cell viability assay

SK-N-AS, SK-N-DZ, NBL-W/S, NBL-W/N, and HeLa cells were trypsinized, collected, counted, and plated (5–6 × 10^3^ SK-N-AS cells per well, 6–7 × 10^3^ SK-N-DZ, 9 × 10^2^ NBL-W/S, 8–9 × 10^3^ NBL-W/N, or 2.5 × 10^3^ HeLa) into 96-well plates and allowed to attach overnight. Five to six wells for each cell line were used as biological replicates in each experiment. Cells were treated with varying concentrations of bare Fe_3_O_4_@TiO_2_ nanocomposites, DOPAC–Fe_3_O_4_@TiO_2_ nanoconjugates, MIBG–Fe_3_O_4_@TiO_2_ nanoconjugates, free DOPAC, free MIBG, DMSO (control for free MIBG experiments), or Na_2_HPO_4_ buffer (10 mM, vehicle control for nanocomposites). In radiosensitization experiments, cells were treated with nanocomposites or nanoconjugates for 1 h, followed by varying doses of ionizing radiation. Cesium source (662 keV) Gamma Irradiator (Gammacell 40, Atomic Energy of Canada Ltd.) was used under the supervision of the Office of Research Safety, Health Physics Services, Northwestern University.

Nanocomposite/nanoconjugate and irradiation treatments were performed in complete media over a period of 72 h. Equivalent “blank” wells without cells, but with an identical concentration of treatment reagents were used as blank controls (n = 5), in order to account for any possible modification of absorbance readout that could occur because of the treatment materials used. After incubation, a tetrazolium compound [3-(4,5-dimethylthiazol-2-yl)-5-(3-carboxymethoxyphenyl)-2-(4-sulfophenyl)-2H-tetrazolium, inner salt] (MTS reagent, Promega, Madison WI) was added as 1/10th of the volume into each well, and the 96-well plates were incubated an additional 2–4 h at 37 °C. Initially, treatment media was removed from both treatment and blank wells before adding MTS and new media; this approach was discontinued when no significant differences in results were observed when MTS was added to wells without any prior manipulation. The latter approach was used subsequently in order to mitigate the risk of inadvertent removal of less adherent cells. Absorbance readings were measured using a SpectraMax M5 (Molecular Devices, Sunnyvale, CA) plate reader at 490 nm. Absorbance values measured for “blank” controls were averaged and subtracted from the treatment values. Resultant absorbance value was divided by the average of the absorbance values for each respective negative control, providing percentage of cell viability as a function of the control (surviving fraction). For radiation sensitization determination, each adjusted absorbance value was divided by the average of each treatment group respective baseline (cells treated with nanocomposites or nanoconjugates but not exposed to radiation) absorbance, to obtain an adjusted percentage of survival, to account for baseline cell death.

### Evaluation of nanocomposite and nanoconjugate uptake by ICP-MS

SK-N-AS and SK-N-DZ cells were trypsinized, counted, and plated (5–8 × 10^5^ of SK-N-AS cells, or 6–10 × 10^5^ SK-N-DZ cells) onto 6-well plates and allowed to attach overnight. Empty wells with identical treatment conditions were also prepared, to act as a control for potential artifacts such as adherence of nanocomposites to the bottom of the wells in absence of cells. Additional wells seeded with an identical number of SK-N-AS or SK-N-DZ cells were used to estimate the final number of cells per well at the conclusion of experiment and to determine background elemental concentrations. Treatments with 250 nM bare Fe_3_O_4_@TiO_2_ nanocomposites, 250 nM DOPAC–Fe_3_O_4_@TiO_2_ nanocomposites, or 250 nM MIBG–Fe_3_O_4_@TiO_2_ nanoconjugates were done in compete media (total volume of media per well was 1 ml) for 1 h at 37 °C; three wells per treatment represented biological replicates. After treatment, the wells were washed 1–3 times with PBS and once with acidic glycine. Finally, 500 μL of 70% HNO_3_ (re-distilled, > 99.999% trace metal basis) was added per well and cells and nanoconjugates were digested for 2 h at room temperature. Samples were then transferred into 15-ml metal-free Falcon tubes, mixed with 10 mL 3% HNO_3_ in ddH_2_O containing 3 ppb ^115^In (as an internal control), and allowed to digest additionally overnight at 70 °C. Samples were evaluated for elemental concentrations of ^47^Ti, ^57^Fe, and ^115^In using an X series II ICP-MS. Ti concentration was used as a proxy for nanoparticle/nanoconjugate concentration. Average background elemental quantity obtained from cell-free nanocomposite-treated blank wells was subtracted from each test sample, to arrive at a final total concentration of nanocomposites taken up by cells. This number was divided by the “end of experiment” cell count to arrive at a Ti concentration per 10^5^ cells. Uptake of bare nanocomposites was used as a standard and uptake of nanoconjugates was expressed as a percentage of the Ti concentration found for uncoated nanocomposites.

### Evaluation of apoptosis/necrosis by flow cytometry

SK-N-AS cells were seeded onto 6-well plates (2 × 10^5^ cells per well) and allowed to attach overnight. Cells treated with 250 nM DOPAC–Fe_3_O_4_@TiO_2_ nanocomposites for one hour as well as untreated controls were either sham irradiated or exposed to 10 Gy of gamma rays from a ^137^Cs irradiator. Cells were then incubated for 24 h, washed with PBS, trypsinized and processed according to manufacturer’s instructions. 10^5^ cells in 100 μL of Annexin-binding buffer were incubated with Annexin-FITC and propidium iodide (PI) for 15 min at room temperature, placed on ice, and immediately evaluated by flow cytometry using a BD LSR Fortessa Analyzer (BD Biosciences, Franklin Lakes, NJ) at the Robert H. Lurie Comprehensive Cancer Center Flow Cytometry Core Facility at Northwestern University. For each biological replicate, 5000 gated events were analyzed.

### Evaluation of 53BP1 foci

A total of 200,000 SK-N-AS cells were seeded onto barrier slides and allowed to attach overnight. Cells in 1 ml full medium were then treated with varying concentrations of DOPAC–Fe_3_O_4_@TiO_2_ nanoconjugates or bare nanocomposites for 1 h. Cells were then irradiated with 2 Gy and incubated for 4 h. Slides were washed with PBS and cells fixed in 3.6% formaldehyde for 10 min at room temperature. Cells were then permeabilized with PBS–Triton (0.2%) for 10 min, rinsed three times with PBS–BSA (1%)—Tween (0.5%) and processed further in the same buffer. Slides were incubated for 1 h with primary antibody against 53BP1 (ab21083—Abcam, Cambridge, UK) used at 1:200 dilution, washed and incubated for 45 min with fluorescent secondary antibody (Alexa Fluor 488—Goat Anti-Rabbit, ab150077, Abcam, Cambridge UK). Nuclei were counterstained with propidium iodide (2.5 μg/ml). Cells were imaged at 40 × magnification with a full field fluorescent Zeiss microscope equipped with a CoolSNAP EZ CCD camera (Photometrics, Tucson AZ, US).

Four experiments were performed with two replicate slides for each treatment condition conducted. The 53BP3 foci in each replicate were counted by a different researcher. Multiple images of each slide were taken, and foci present in at least 100 cells were counted for each treatment group. All experiments were pooled as indicated (slide numbers ranged between 3 and 7) and statistics generated from the pooled data.

### Cryogenic X-ray fluorescence microscopy (Cryo-XFM)

SK-N-AS and SK-N-DZ cells were seeded on 1.5 mm × 1.5 mm Si_3_N_4_ windows overnight (Silson, UK), then treated either with 4.24 µM MIBG (with a resultant 0.05% DMSO concentration), 25.44 µM MIBG (final 0.30% DMSO), 60 µM MIBG (final 2% DMSO), or DMSO control (0.30% DMSO) in 50 µL of full media for 90 min. In nanocomposite treatment experiments, cells were treated with MIBG–Fe_3_O_4_@TiO_2_–B-loop nanoconjugates (carrying an equivalent of 60 µM of MIBG) for 90 min in serum-free DMEM. In addition, another set of cells grown on Si_3_N_4_ windows were exposed to 250 nM MIBG–Fe_3_O_4_@TiO_2_, 250 nM DOPAC–Fe_3_O_4_@TiO_2_, or 250 nM of bare (phosphate covered) Fe_3_O_4_@TiO_2_ nanocomposites for 60 min in 50 µL of full media. The windows were washed twice in a Tris glucose buffer (261 mM glucose, 9 mM acetic acid, 10 mM Tris buffer, pH 7.4) and plunge-frozen in liquid ethane using a FEI Vitrobot Mark IV. Frozen hydrated cells were imaged with visible light on a Nikon microscope equipped with an Instec CLM77KCryo-LM stage in order to evaluate the quality of each sample with regard to ice accumulation as well as cell density and distribution.

X-ray fluorescence imaging was done with several different instruments under different conditions. A beam spot size of about 300 or 600 nm was used at the sector 2-ID-D at APS at ANL in combination with a cryo-jet; while the Bionanoprobe at sector 21 LS-CAT was used with a beam spot size 85 nm, and the samples were maintained in vacuum at liquid nitrogen temperature. High-resolution elemental maps were obtained at different angles, allowing subsequent tomographic reconstruction. A monochromatic 10 keV X-ray beam was used and the cells were scanned in “continuous” (fly-scan) mode. Step scans for an area of interest were also done at a step size of 80 nm and per-pixel dwell time of 3 s. To minimize background noise, a Gaussian smoothing filter (*σ* = 2/3) was applied to the images in the figures presented.

For tomographic reconstruction, scans were done at multiple angles (3-degree increments, total angular range of 138 degrees); reconstructions were performed in Mathematica 9.0 (Wolfram Research, Champaign, IL). In order to circumvent misalignment of particles as a result of sample movement during scanning, the “displacement” of particles with clear projections (Cl, I, K, P and S) was analyzed, and the average shift along the x and y axis of these particles was calculated. Correction of any displacement of the tested particles was achieved by applying this calculated shift to all the particles, hence any misalignment was adjusted. Tomographic reconstruction was attained by implementing a modified simultaneous iterative reconstruction technique (SIRT) rather than filtered back projection (FBP) since the later yields poor images owing to the limited number of projections. Use of SIRT was described previously with the final visualization of elemental signals of interest using Avizo (FEI, Burlington, MA) (Vo et al. 2014). Elemental concentration data were extracted for each pixel of the 2D images (elemental quantification, per-pixel fitting) using the MAPS program (Vogt et al. 2003).

### Statistical analyses

All comparisons were performed using Student’s *T*-test at a significance level of 0.05. Data points in all figures correspond to mean ± standard deviation.

## Supplementary Information

**Additional file 1.** Additional tables and figures.

**Additional file 2.** Video of the rotation of the cell shown in Figure 3.

## Data Availability

All data generated or analyzed during this study are included in this published article and its Additional file 1 and Additional file 2.

## Abbreviations

53BP1: P53-Binding Protein 1
AFM: Atomic force microscopy
APS: Advanced Photon Source
ANL: Argonne National Laboratory
DNA: Deoxyribonucleic acid
DOPAC: 3,4-Dihydroxyphenylacetic acid
EDS: Energy-dispersive X-ray spectroscopy
EDC: 1-Ethyl-3-(3-dimethylaminopropyl) carbodiimide hydrochloride
EGF: Epidermal growth factor
EGFR: Epidermal growth factor receptor
Fe_3_O_4_: Iron oxide
Fe_3_O_4_ @ TiO_2_: Iron oxide core, titanium dioxide shell nanocomposite
ICP-MS: Inductively coupled plasma-mass spectrometry
IRENI: Infrared environmental imaging
MIBG: Metaiodobenzylguanidine
MTS: 3-(4,5-Dimethylthiazol-2-yl)-5-(3-carboxymethoxyphenyl)-2-(4-sulfophenyl)-2H-tetrazolium
MYCN: N-myc proto-oncogene protein
NRER: Nanocomposite radiation enhancement ratio
TEM: Transmission electron microscope
TiO_2_: Titanium dioxide
S-type: Epithelial morphology in neuroblastoma
SER: Sensitizer enhancement ratio
STEM: Scanning transmission electron microscope
RAS–MAPK: RAS–mitogen-activated protein kinase
ROI: Region of interest
XFM: X-ray fluorescence microscopy
