# Supplementary material for: Development of Fe3O4 core–TiO2 shell nanocomposites and nanoconjugates as a foundation for neuroblastoma radiosensitization
Source: Cancer Nanotechnol. 2021 May 14;12(1):12. doi: 10.1186/s12645-021-00081-z (PMC8550682; doi:10.1186/s12645-021-00081-z)
Supplement: Supplementary file 1 — Additional file 1. Additional tables and figures. [file 12645_2021_81_MOESM1_ESM.docx]

**Additional Data:**

**Development of Fe_3_O_4_ core-TiO_2_ shell nanocomposites and nanoconjugates as a foundation for neuroblastoma radiosensitization**

William Liu^1^, Salida Mirzoeva^1^, Ye Yuan^1^, Junjing Deng^2^, Si Chen^3^, Barry Lai^3^, Stefan Vogt^3^, Karna Shah^1^, Rahul Shroff^1^, Reiner Bleher^4^, Qiaoling Jin^2^, Nghia Vo^5^, Remon Bazak^8^, Carissa Ritner^1^, Stanley Gutionov^1^, Sumita Raha^1^, Julia Sedlmair^6^, Carol Hirschmugl^6,7^, Chris Jacobsen^2,3^, Tatjana Paunesku^1^, John Kalapurkal^1^, and Gayle E. Woloschak^1*^

**Affiliations:**

1. Department of Radiation Oncology, Northwestern University, Chicago IL 60611, US
2. Department of Physics and Astronomy, Northwestern University, Evanston IL, 60208, US
3. X-ray Science Division, Argonne National Laboratory, Argonne IL, 60439 US
4. Chemistry of Life Processes Institute, Northwestern University, Evanston IL, 60208, US
5. Diamond Light Source Ltd., Harwell Science and Innovation Campus, Didcot OX11 0DE, UK
6. Synchrotron Radiation Center, 3731 Schneider Drive, Stoughton, WI 53589-3097, US
7. Physics Department, University of Wisconsin-Milwaukee, Milwaukee, WI 53211
8. Department of Otorhinolaryngology, University of Alexandria, Faculty of Medicine, Alexandria, Egypt

[*g-woloschak@northwestern.edu](mailto:*g-woloschak@northwestern.edu) (corresponding author)


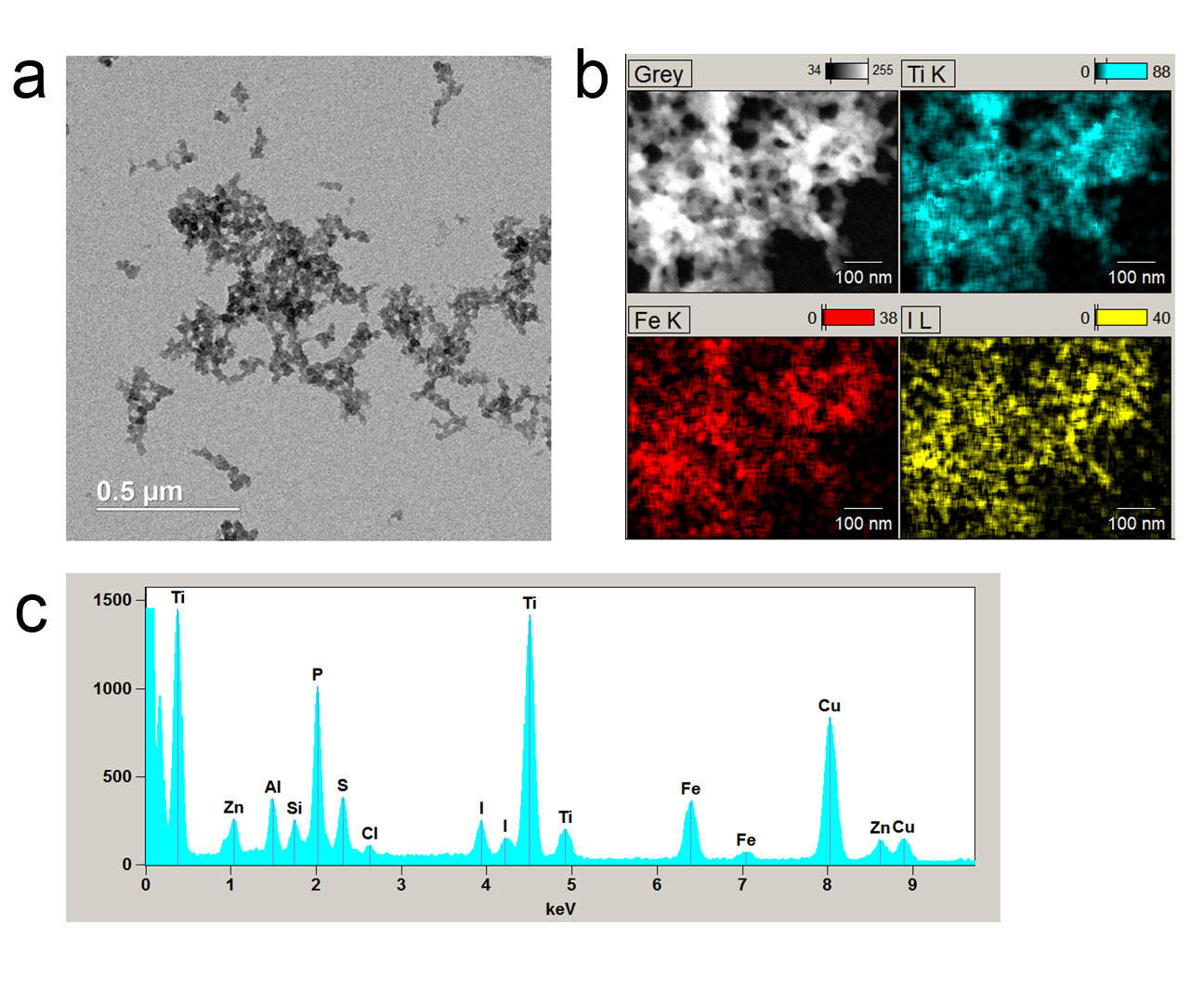


Additional file 1: Figure S1. Imaging of Fe_3_O_4_@TiO_2_ covered with MIBG by EDS-STEM

(a) STEM (bright-field) image of MIBG-Fe_3_O_4_@TiO_2_ nanoconjugates, showing a degree of polydispersity and aggregation of nanoconjugates dried in air on a carbon coated TEM grid. (b) EDS-STEM imaging shows the elemental overlay (Z-contrast) of Ti, Fe, and I signal from nanoconjugates in a subregion from (a). This elemental map is demonstrating the co-localization of I (originating from MIBG) with Ti and Fe signals originating from the nanocomposite. (c) EDS generated elemental spectra from the area of interest in (b). Please note that in order to produce a molecule containing MIBG on one side and a catechol on the other, we created a peptide bond between MIBG and DOPAC by a chemical reaction facilitated by Sulfo-NHS ester following the protocol provided by Thermo-Fisher. Elements in this spectrum other than Yi, Fe and I come from chemicals used for these chemical reactions. Images were obtained at 200 kV.


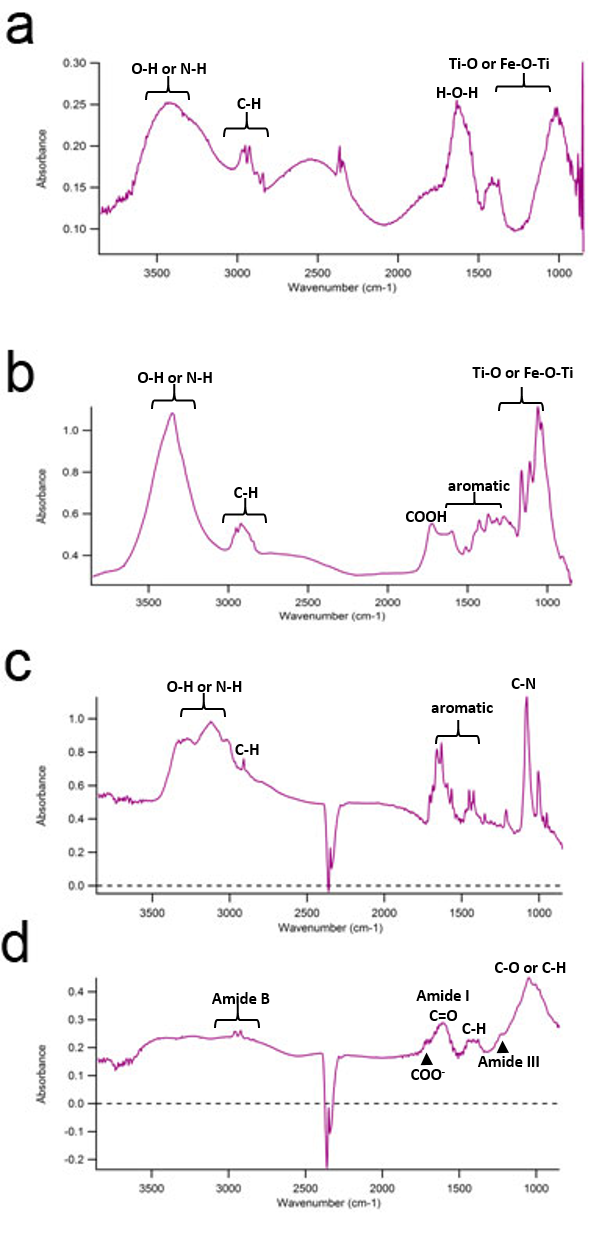


Additional file 1: Figure S2. Infrared spectroscopy of nanocomposites and their components at different stages of preparation

Infrared spectroscopy chemograms of nanoconjugate aliquots separated from the remainder of the synthesis mixture during the process of conjugation. Specifically – the process of preparation began with the Fe_3_O_4_@TiO_2_ nanocomposites as synthesized, next – their surface was coated with DOPAC and finally DOPAC served as an anchor for peptide bond formation with MIBG. FTIR spectroscopy was done using the IRENI instrument.

a) Fe_3_O_4_@TiO_2_ nanocomposites after dialysis. In the Fe_3_O_4_@TiO_2_ sample the peaks that appear at ~1000 cm^-1^ and 1415 cm^-1^ could be associated with the presence of stretching vibrations of Ti-O and Fe-O-Ti bonds (Nemati et al. 2015 ; Xin et al. 2014). The bands at ~1640 cm^-1^ belong to H-O-H bending vibration, indicating that several OH groups are present in the sample as a result of TiO_2_ coating or vibration of water molecules adsorbed on the surface of NP.

The peaks in the range ~2960 cm^-1^ can be attributed to the C-H bending vibrations and C-O stretching.

b) DOPAC- Fe_3_O_4_@TiO_2_ nanocomposites after dialysis. DOPAC- Fe_3_O_4_@TiO_2_ nanocomposites showed several peaks in the range of 1066, 1123, 1169 cm^-1^ which possibly originated from Ti-O and Fe-O-Ti stretching bonds, while bonds ~1370 and 1726 cm^-1^ can be ascribed to C-H vibration and carboxyl COOH bonds, respectively. The band at ~2900 cm^-1^ can be attributed to the C-H bending vibrations, while band at ~3350 is associated to N-H stretching vibrations.

c) MIBG. FTIR spectra of the MIBG: bands at ~1075 and ~1415 cm^-1^ could be associated possibly to CH-I vibrations. Bands at ~1443, 1632 and 1660 cm^-1^ are related to aromatic ring vibrations. Band at ~2896 cm^-1^ is associated to C-H stretching vibration, and band at ~3113 cm^-1^ is associated with N-H vibration.

d) MIBG-(DOPAC)- Fe3O4@TiO2 nanocomposites after dialysis. MIBG-(DOPAC)- Fe_3_O_4_@TiO_2_ nanocomposites IR spectra showed the characteristic bands of C-H skeleton and C-O stretching vibration at ~1047 cm^-1^  and C-H deformation vibration at ~1405 cm^-1^. Broadband around 1610 cm^-1^ indicate the C=O vibration of Amide I in the newly generated peptide bond. Bands at 2915 and 2952 cm^-1^ represent C-H asymmetric stretching vibrations and Amide B vibration. A week band at ~1238 cm^-1^ possibly indicates Amide III vibration. Finally a small peak at ~1706 cm^-1^  could be assigned to COO- vibration of carboxyl groups.


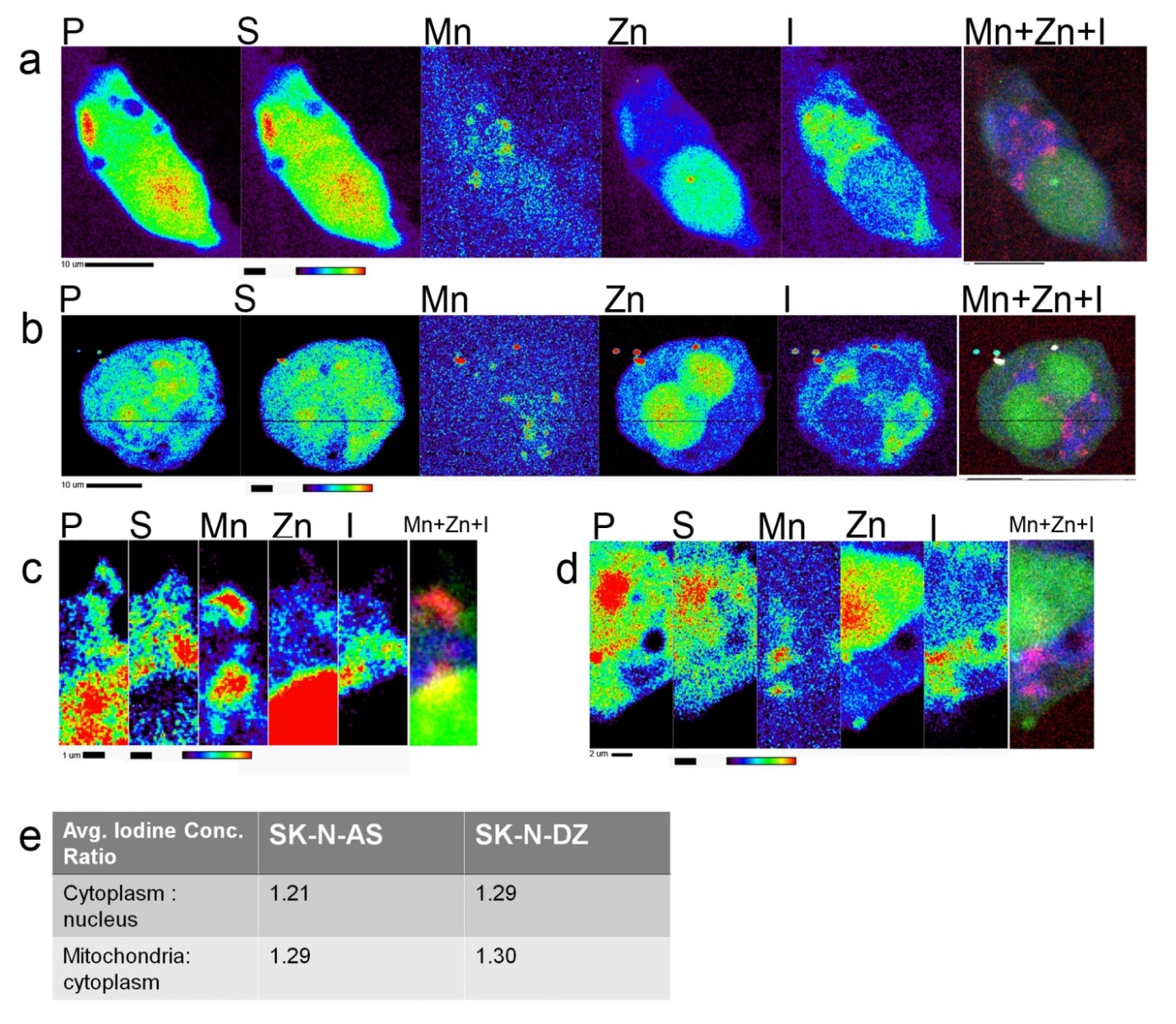


Additional file 1: Figure S3. Evaluation of Distribution of Free MIBG in Neuroblastoma Cells Evaluated by Cryo-XFM

(a) SK-N-AS cells treated with MIBG (60 µM) show cytoplasmic (outlines of cytoplasm can be considered to be outlines of S signal) distribution of MIBG (detected through the presence of I), with some overlap of I signal with mitochondria (enriched for the presence of Mn), but exclusion from the nucleus (the area overlapping with the strongest Zn signal). The co-localization image: Mn: Red, Zn: Green, I: Blue. (b) A similar result was observed in SK-N-DZ cells. (c) A smaller step scan (higher detail image) of a region of interest from the cell shown in (a) with cytoplasmic distribution of I, with apparent exclusion from the nucleus. (d) A smaller step scan of a region of interest from the cell shown in (b). (e) A table of iodine concentration ratios for the cytoplasm: nucleus or mitochondria: cytoplasm. 21 or 29% higher concentration of iodine is observed in the cytoplasm compared to the nucleus of the SK-N-AS or the SK-N-DZ cells, respectively. Mitochondria had a 29% or 30 % greater concentration of iodine than the cytoplasm in the SK-N-AS or the SK-N-DZ cells, respectively. Scale bar and elemental concentration indicator (black – no signal to red – highest signal) are located under each image.


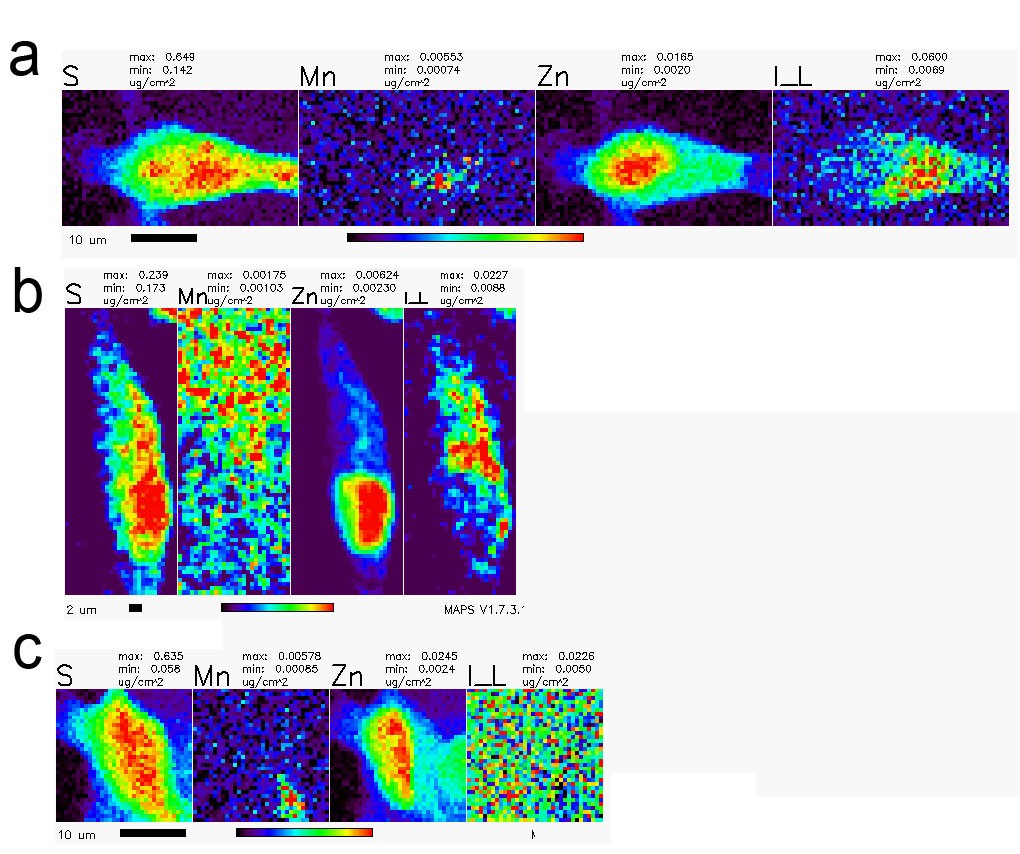


Additional file 1: Figure S4. Lower resolution cryo-XFM scans of AS cells at lower MIBG concentrations and vehicle (DMSO) control.

A) An SK-N-AS cells treated with 25.44 µM MIBG b) or 4.24 µM MIBG c) or DMSO (.30%) alone as a control. a-b) Cells given decreased concentration of MIBG show similar pattern of I distribution as cells treated with higher concentrations of MIBG. No I signal can be noticed in DMSO control where “salt and pepper” pixel pattern indicates only background pixel intensity values. Scale bar and elemental concentration indicator (black – no signal to red – highest signal) are located under each image.


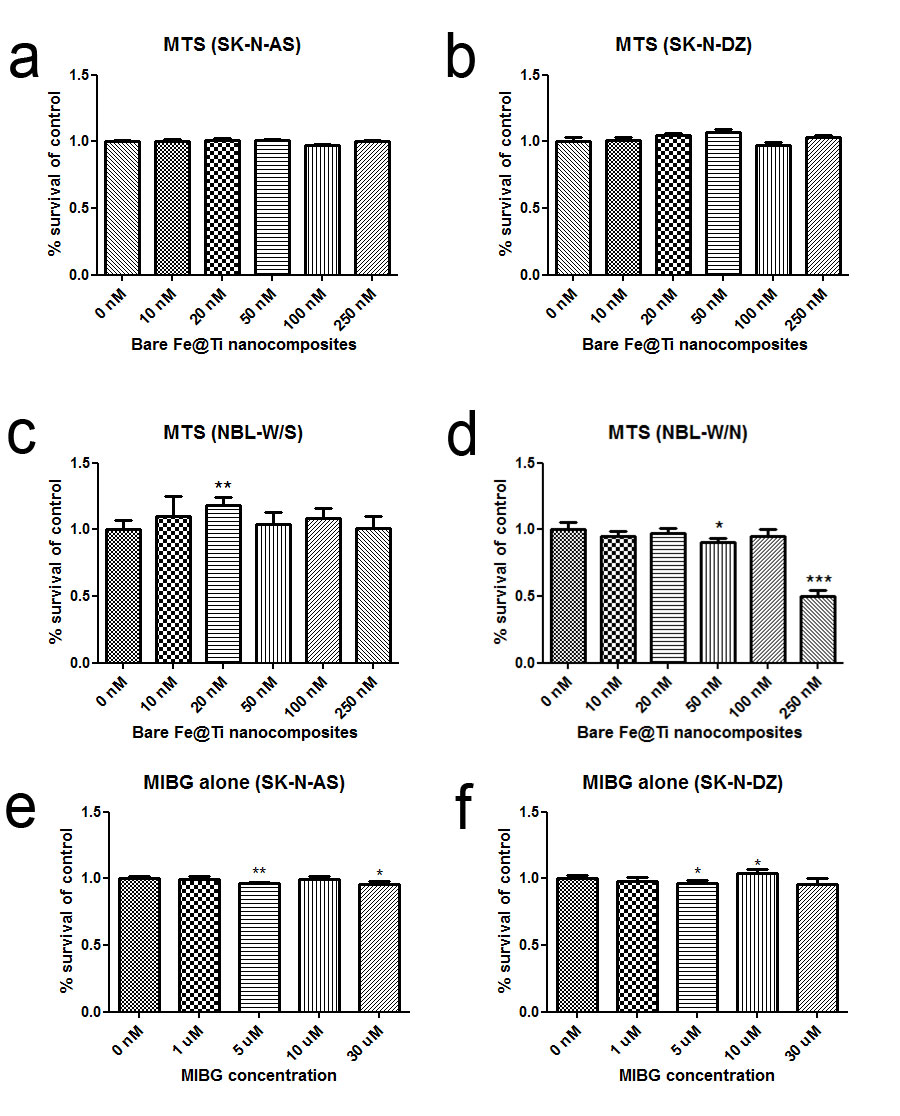


Additional file 1: Figure S5. Survival of neuroblastoma cells treated with Fe_3_O_4_@TiO_2_ nanocomposites

(a) SK-N-AS cells treated with increasing doses of bare surface Fe_3_O_4_@TiO_2_ nanocomposites. No significant decrease in survival was observed. (b) a similar result was observed in SK-N-DZ cells and (c) NBL-W/S cells also exhibited a similar response, although at lower concentrations, there was a significant increase in cell proliferation in response to bare nanocomposite treatment. (d) NBL-W/N cells exhibited a significant decrease in survival after nanocomposite treatment, particularly at 250 nM concentration. (e-f) SK-N-AS and SK-N-DZ cells treated with varying concentrations of free MIBG. The effect of MIBG on cell survival was minimal. Data points presented are an average of 5 biological replicates, and this dataset is representative of a minimum of two independent experiments. *<0.05 significance level, ** : <0.01 significance level, *** <0.001 significance level. Error bars indicate mean ± SD.


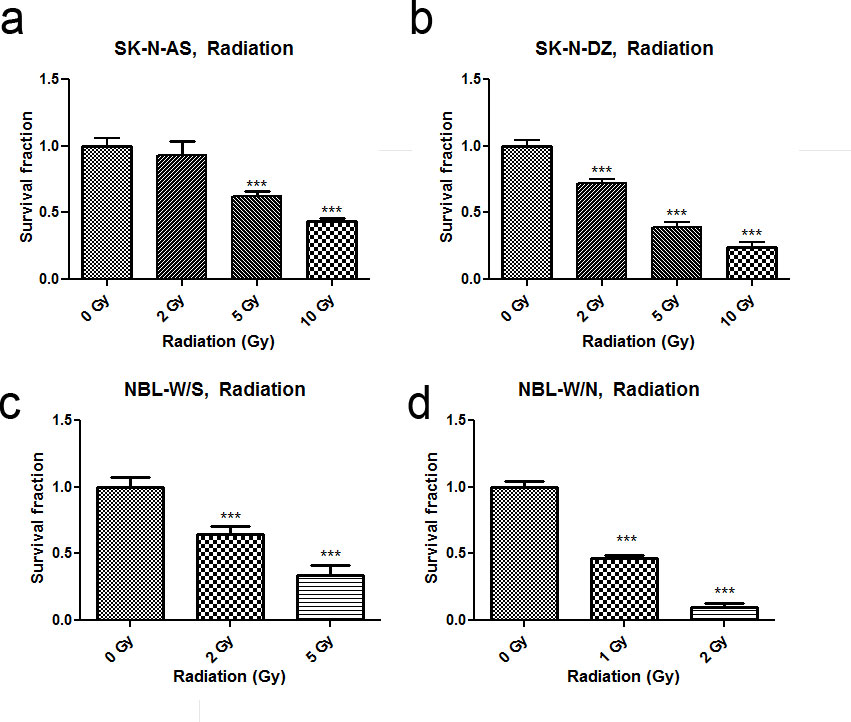


Additional file 1: Figure S6. Survival of different neuroblastoma cell lines in response to ionizing radiation evaluated by MTS assay

(a) SK-N-AS (b) SK-N-DZ (c) NBL-W/S (d) NBL-W/N. * <0.05 significance level, ** :<0.01 significance level, *** <0.001 significance level. Datapoints presented are an average of at least 5 biological replicates, and are representative of at least three independent irradiation experiments followed by an MTS assay as detailed in the Methods. Error bars indicate mean ± SD.


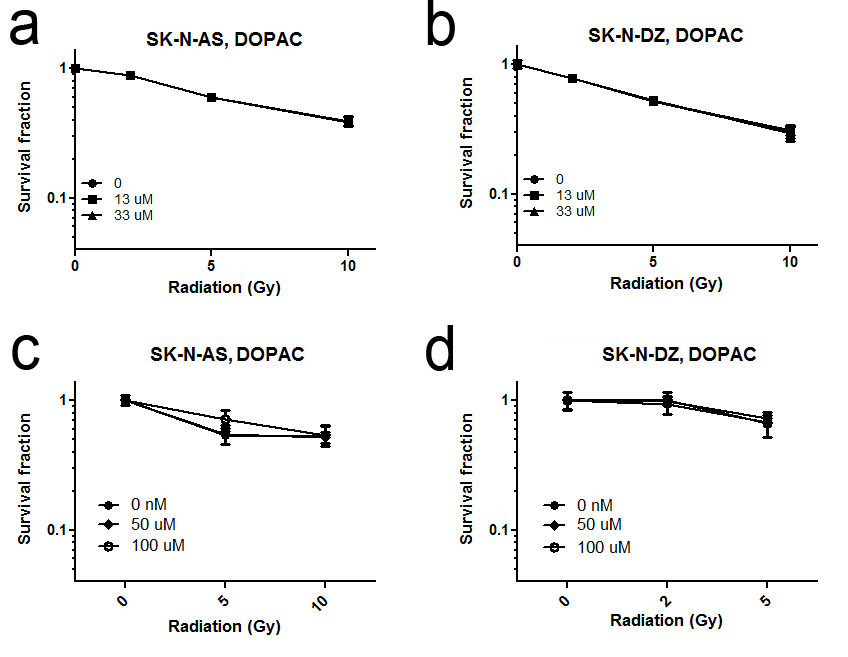


Additional file 1: Figure S7. No radiosensitization is observed in SK-N-AS and SK-N-DZ cells irradiated in the presence of DOPAC molecule

(a) SK-N-AS and (b) SK-N-DZ cells irradiated with different doses of x-rays were previously treated with DOPAC. Molecular DOPAC concentrations were equivalent to the concentration of DOPAC estimated to be bound to the surface of the nanocomposite. No sensitization was found in either SK-N-AS or SK-N-DZ cell lines (n = 5). This trend was unchanged at higher DOPAC concentrations (c,d) (n = 3). Error bars indicate mean ± SD.

|  | **Bare NC** | **MIBG NC** | **DOPAC NC** |
| --- | --- | --- | --- |
| **ZP (mV)** | -37.1 ± 1.91 | -40.887 ± 1.85 | -33.367 ± 0.71 |

Additional file 1: Table S1. Zeta potentials of nanocomposites and nanoconjugates

Bare nanocomposites, DOPAC- Fe_3_O_4_@TiO_2_ nanocomposites, and MIBG- Fe_3_O_4_@TiO_2_ nanoconjugates were diluted 1:100 in filtered 10 mM NaCl and Zeta potentials measured at 25°C, as an average of three measurements.

| **Nanosight (buffer)** | **Bare NC** | **MIBG NC** | **DOPAC NC** |
| --- | --- | --- | --- |
| **DMEM** | 278 ± 117 | 272 ±110 | 291 ±117 |
| **ddH_2_O** | 269 ± 182 | 194 ± 98 | NA |

Additional file 1: Table S2. Nanocomposite and nanoconjugate sizing by Nanosight

Bare nanocomposites, MIBG- Fe_3_O_4_@TiO_2_ nanoconjugates, and DOPAC- Fe_3_O_4_@TiO_2_ nanocomposites were diluted 1:100 in DMEM or ddH_2_O and sizing evaluated on a Nanosight LM10-HS. Due to polydispersity and non-spherical shape of aggregates, sizing data obtained were not deemed particularly reliable.

REFERENCES:

Nemati F, Heravi MM, Elhampour A (2015) Magnetic nano-Fe3O4@TiO2/Cu2O core-shell composite: an efficient novel catalyst for the regioselective synthesis of 1,2,3-triazoles using a click reaction RSC ADVANCES 5:45775-45784 doi:10.1039/c5ra06810j

Xin T, Ma M, Zhang H, Gu J, Wang S, Liu M, Zhang Q (2014) A facile approach for the synthesis of magnetic separable Fe3O4@TiO2, core shell nanocomposites as highly recyclable photocatalysts APPLIED SURFACE SCIENCE 288:51-59 doi:10.1016/j.apsusc.2013.09.108
